# Untangling the Mechanisms in Magneto‐Electrocatalytic Oxygen Evolution

**DOI:** 10.1002/smll.202412852

**Published:** 2025-05-02

**Authors:** Amy Radford, Dorottya Szalay, Qiming Chen, Mengfan Ying, Mingyu Luo, Xuelei Pan, Michail Stamatakis, Yiyang Li, Chen Wu, Shik Chi Edman Tsang

**Affiliations:** ^1^ Inorganic Chemistry Laboratory Department of Chemistry University of Oxford Oxford OX1 3QR UK; ^2^ School of Materials Science and Engineering State Key Laboratory of Silicon and Advanced Semiconductor Materials Zhejiang University Hangzhou 310027 China

**Keywords:** electrocatalysis, lorentz forces, magnetism, magneto‐electrocatalysis, mechanisms, spin‐polarization, water‐splitting

## Abstract

External magnetic fields emerge as a promising method for enhancing the electrocatalytic oxygen evolution reaction (OER), yet the underlying magneto‐electric (ME) mechanisms are not well understood. The slow kinetics of OER make it a key challenge in electrocatalytic water‐splitting, a promising technique for sustainable H_2_ fuel production. Herein, a systematic approach is presented to analyzing the ME mechanisms governing OER, using metallic‐plate (Ni foam, Ni sheet, and Pt sheet) and powder‐based (Co_3_O_4_/BaFe_12_O_19_ on carbon paper) electrodes. Through controlled experiments using varying magnetic field strengths and orientations, Lorentz force and spin‐polarization mechanisms are separated. For metallic electrodes, the effects are orientation‐dependent, indicating domination by Lorentz force. Magnetic flux density about the electrode surface is shown to govern the Lorentz force behavior. Interestingly, a “pseudo” effect is discovered which results from the relative position of the reference electrode, highlighting the importance of experimental design. The Co_3_O_4_ systems display minimal orientation dependence, indicating spin‐polarization domination. Introducing BaFe_12_O_19_ as a magnetic co‐catalyst further amplifies the ME effect, marking the first demonstration of magnetic co‐catalyst enhancement in magneto‐electrocatalysis. This work provides key insights into ME mechanisms, linking electrode composition, magnetism, and geometry to performance, offering new pathways for optimizing future magneto‐electrocatalytic systems.

## Graphical abstract

Magnetic fields can enhance the oxygen evolution reaction in electrocatalytic water‐splitting for green‐H_2_. Yet, commercialization cannot be realized until the underlying magneto‐electric mechanisms are understood. This work uses reactor orientation to untangle these mechanisms. Lorentz force is shown to control metallic plates, while spin‐polarization controls Co_3_O_4_ powder. A new ‘pseudo’ mechanism is also exposed which depends on the reference electrode position.

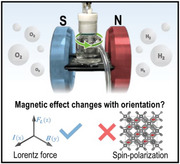

## 1 Introduction

Static magnetic fields can be exploited to enhance electrocatalytic water‐splitting (EWS). They have attracted increasing attention due to the ability of magneto‐electric (ME) mechanisms to simultaneously couple mechanical and electron transfer effects, using relatively low energy input.^[^ ^1^, ^2^, ^3^ ^]^ However, reports on magnetic field effects vary widely, with some studies demonstrating significant enhancements of up to 650% in EWS performance, and others reporting neutral or even negative effects.^[^ ^4^, ^5^ ^]^ Such discrepancies highlight the need for a deeper understanding of the ME mechanisms that control EWS under magnetic fields.

After progression in the field, work on optimizing magneto‐electrocatalytic water‐splitting toward industrial scale‐up can begin. Whilst the specific system design cannot yet be perceived, the two main features of the technique – magnets and EWS – have already proven to be industrially‐feasible.^[^ ^6^, ^7^ ^]^ In the general OER field, it's possible that magnetic fields will become a standard consideration for performance enhancement alongside other external fields and energy sources, such as light, heat, and ultrasonic waves.^[^ ^8^, ^9^ ^]^

The OER and hydrogen evolution reactions are the two half‐reactions that occur in overall EWS. As a four‐electron process with sluggish kinetics and high overpotential requirements, OER generally limits the efficiency of EWS systems.^[^ ^10^, ^11^ ^]^ Improving the performance of OER catalysts and hence, EWS, is vital in the pursuit of affordable green hydrogen (H_2_) fuel. Green H_2_ offers a sustainable alternative to traditional fossil fuels due to its high energy density and environmentally clean combustion, producing only water as a byproduct.^[^ ^12^ ^]^ Among the methods for green H_2_ production, EWS driven by renewable energy is considered as one of the most sustainable approaches.

Electrocatalytic reactions consist of three major steps: i) mass transport of reagents (i.e., diffusion and adsorption), ii) reaction on the electrode surface (i.e., electron transfer and bond breaking/forming), and iii) mass transport of products (i.e., desorption and diffusion).^[^ ^13^ ^]^ The ME mechanisms are linked to these steps and tend to manifest as: magnetohydrodynamics (MHD) (affecting steps i, iii), magnetoresistance (MR) (steps i, ii, iii), and promoted triplet oxygen formation (POF) (step ii).^[^ ^14^ ^]^ These mechanisms primarily originate from Lorentz force effects and spin‐polarization and, to a lesser extent, Kelvin force and Maxwell stress. The interplay of these mechanisms depends on several factors, including electrode composition, electrode geometry, electrode magnetization, reactor design, magnetic field orientation, and magnetic field strength.^[^ ^15^, ^16^ ^]^

Lorentz force acts upon moving charges under a magnetic field and is described by Equation (1):

(1) $$F_{L}=j\times B$$

where *j* is the current density and *B* is an effective magnetic field.^[^ ^5^ ^]^ The direction of Lorentz force is perpendicular to the co‐plane of *B* and *j* – described by Fleming's left‐hand rule – which makes it highly orientation‐dependent.

Originating from the inter‐electrode and intra‐electrode electric fields (*i_inter_* and *i_intra_*, respectively), Lorentz force can cause convection of ions in the electrolyte – creating an MHD effect.^[^ ^17^ ^]^ MHD can affect mass transport by changing the diffusion layer thickness and by influencing the O_2_ bubble release from the electrode surface.^[^ ^5^, ^17^, ^18^, ^19^, ^20^ ^]^ In particular, when *B* and *i_inter_* are perpendicular, vertical Lorentz forces can promote (upward force) or inhibit (downward force) bubble release depending on their alignment with natural buoyancy forces.^[^ ^5^, ^20^ ^]^ Promoted bubble‐release increases active site availability. When *B* and *i_inter_* are parallel, Lorentz force is minimal. However, micro‐convection can be observed where the electric field is bent at the electrode edges, bulges in the surface, and around adsorbed bubbles.^[^ ^17^ ^]^

Hall effects can be induced when Lorentz force interacts with *i_intra_*. Resulting in an accumulation of charge at one side of a material and increased resistance of charge flow – known as positive magnetoresistance (pMR).^[^ ^21^ ^]^

Spin‐polarization describes the alignment of electron spins in a material containing unpaired electrons and can be quantified by magnetization. The electron transport properties of a material can be affected upon magnetization, which is described by magnetoresistance. Negative magnetoresistance (nMR) can result from the creation of spin channels, which facilitate electron transfer through decreased spin‐disorder scattering.^[^ ^4^, ^22^ ^]^ Alternatively, pMR can arise when a magnetic field induces spin mixing through the Zeeman effect, which can lead to increased spin‐disorder scattering.^[^ ^23^, ^24^ ^]^

In POF, spin‐polarized active sites align the spins of reaction intermediates, favoring the formation of molecular ground‐state triplet oxygen (^3^O_2_) which contains parallel‐aligned electrons in its frontier π* orbitals.^[^ ^25^ ^]^ Ren et al. combined computational and experimental methods to demonstrate this enhancement mechanism on a ferromagnetic CoFe_2_O_4_ catalyst.^[^ ^26^ ^]^

While Kelvin force and Maxwell stress are also discussed in magneto‐electrocatalysis, their influence is typically minimal in common water‐splitting electrolytes (i.e., KOH, KCl, and H_2_SO_4_) due to the scarcity of paramagnetic species.^[^ ^14^ ^]^ Under extremely high magnetic fields, paramagnetic ^3^O_2_ may succumb to Kelvin force effects. This pushes the molecules toward areas of high magnetic flux density, leading to restricted product diffusion.^[^ ^27^ ^]^

Targeting the field's limited understanding of ME mechanisms and their interplay in OER, this work exemplifies a thorough analysis of the contributing mechanisms in two distinct electrode systems: metallic plate‐shaped electrodes (Ni foam, Ni sheet, and Pt sheet), and powder‐based electrodes (Co_3_O_4_/BaFe_12_O_19_ powder on carbon paper). Building on earlier studies using similar catalysts.^[^ ^4^, ^5^, ^20^, ^26^, ^28^, ^29^ ^]^ Pulsed magneto‐chronoamperometry (PMCA) experiments have been employed to quantify the magnetic effects under varying MF strengths and orientations. The aim of this work is to improve on many of the existing works that neglect the impact of magnetic field orientation or overlook experimental factors, such as reactor geometry and reference electrode positioning.

Lorentz force and spin‐polarization contributions have been successfully separated by studying orientation‐dependence. Lorentz force is identified as the main source of magnetic effects for the metallic electrodes and is found to be mediated by the surface magnetization of the electrode. Spin‐polarization controls the powder‐based Co_3_O_4_ on carbon paper electrodes. These magnetic effects have been enhanced using ferromagnetic BaFe_12_O_19_ as a co‐catalyst, by increasing local magnetic flux density around the Co_3_O_4_ catalyst.^[^ ^30^ ^]^ This represents the first reported application of a magnetic co‐catalyst in electrocatalysis, demonstrating a novel strategy for enhancing ME effects without altering the primary catalyst composition.

Notably, an important discovery has been made that must be considered in all future magneto‐electrocatalytic experiments using a reference electrode (RE). A “pseudo” magnetic effect has been identified in Lorentz force‐controlled systems. Electrode polarization and edge‐MHD create differences across the horizontal *x–y* plane of the WE, and the resulting pseudo effect depends on the relative RE position during the measurement.

This study provides a framework of systematic analysis for identifying and optimizing ME mechanisms in magneto‐electrocatalytic systems. By linking electrode composition, magnetism, and geometry to specific ME effects, this work contributes to the rational design of future magnetic field‐assisted electrocatalytic systems.

## 2 Results and Discussion

**Figure 1:**
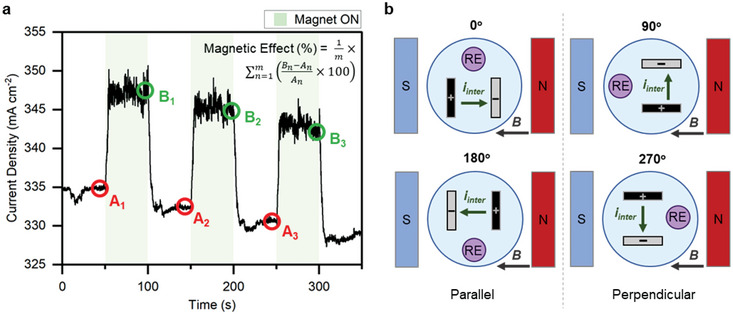
a) Percentage magnetic effect calculation from PMCA measurements. Conditions: 1 cm^2^ of WE and of CE submerged in 20 mL electrolyte (1 m KOH), held in a 30 mL water‐cooled (20 °C) reactor. b) Schematics of the four electrode orientations with respect to the applied magnetic field: 0°, 90°, 180°, and 270° where the WE is black, CE is gray, and RE is purple.

The influences of magnetic field strength and orientation on the ME effects of two series of electrodes have been investigated. PMCA (**Figure** **1**a) experiments were conducted to study magnetic effects on the performance of the working electrode (WE) with a Pt counter electrode (CE) and a Hg/HgO RE in 1 m KOH. A photo of the experimental setup can be found in Figure S1 (Supporting Information). Using an electromagnet, 50‐second magnetic field pulses were applied in intervals up to 800 mT. The reactor was rotated into four positions with the angle between the electrodes and the magnetic field set at 0° (standard), 90°, 180°, and 270° (Figure 1b). The magnetic effect is quantified by the average percentage change in current density between the pre‐application of magnetic field (i.e., A_n_ in Figure 1a) and at ≈50 s of magnetic field application (i.e., *B*_n_ in Figure 1a). Where an increase in current density defines a “positive effect” and a decrease defines a “negative effect”.

PMCA analysis most accurately represents the ME effects because changes in current density can be observed in real‐time, and the constantly applied potential creates a stable system. The electrocatalytic performance data is coupled with magnetization and MR analysis to evaluate the ME mechanisms dominating each electrode system.

### 2.1 Lorentz Force Domination in Metallic Electrodes: Ni Foam, Ni Sheet, and Pt Sheet

Ni and Pt metals form simple OER electrodes which ease the interpretation of the ME mechanisms at play. The transition metal surfaces are hydroxylated in the alkaline reaction conditions. Then, under reaction conditions, the hydroxides are oxidized to form molecular O_2_ via oxyhydroxides.^[^ ^31^, ^32^, ^33^ ^]^ Direct comparisons of electrode magnetism can be made between ferromagnetic Ni sheets and paramagnetic Pt sheets; with any ferromagnetic effects being corroborated by Ni foam. The different structures and densities of Ni foam and Ni sheet make for an interesting analysis on the impacts of morphology and absolute electrode magnetization.

Further, a “pseudo” ME effect due to relative RE position is uncovered in this system which may be present but, as yet unidentified, in similar ME systems in the literature.^[^ ^16^, ^27^, ^34^ ^]^

#### 2.1.1 Electrode Characterization

**Figure 2:**
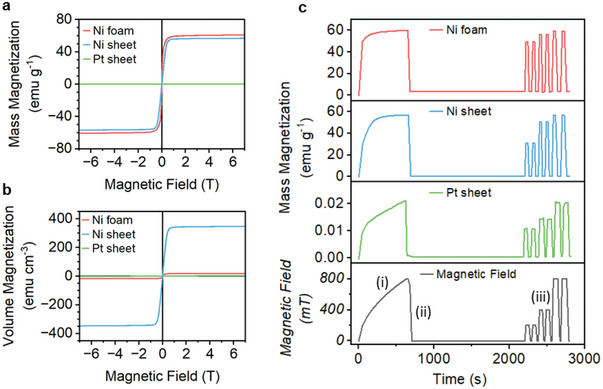
Magnetization versus magnetic field plots for the Ni foam, Ni sheet, and Pt sheet: a) mass magnetization and b) volume magnetization. c) Plots of mass magnetization versus time under a sequence of magnetic field strengths: i) slow field ramp up to 800 mT, ii) field removal to 0 mT, iii) ≈50 s field pulses between 0 mT and 200, 400, and 800 mT. These plots demonstrate the magnetic remanence of each electrode, where the ≈50 s pulses mimic the PMCA experiments.

XRD data taken from the Ni and Pt electrodes show crystalline structures (Figure S2, Supporting Information). Magnetometry measurements (**Figure** **2**a) show clear ferromagnetic properties for the Ni foam and Ni sheet, which respectively exhibit low coercivities of 1.33 and 0.44 mT (Figure S3, Supporting Information), magnetization at 800 mT (*M_800_*) of 58.7 and 55.4 emu g^−1^, and saturation magnetization (*M_s_*) of 60.8 and 56.7 emu g^−1^. The Pt sheet shows paramagnetic character, with an *M_800_* of 0.023 emu g^−1^. Volume magnetizations are shown in Figure 2b which allows comparison between the electrodes’ total magnetizations, since the submerged volume of the electrodes is kept consistent at 0.1 cm^3^ but the mass varies. The volume *M_800_* of the Ni sheet (339.2 emu cm^−3^) is much larger than Ni foam (17.2 emu cm^−3^) due to differences in density; the Pt sheet has a volume *M_800_* of 0.6 emu cm^−3^. Magnetic retentivity is shown in Figure 2c, where the mimic PMCA pulses, performed using magnetometry, show low retentivity of magnetization for all the electrodes. This makes them ideal for quick magnetic pulses during electrocatalysis.

#### 2.1.2 OER in a Magnetic Field

Initial experiments on magnetic effects were conducted in the 0° orientation. The Ni electrodes consistently show a positive effect and so the possible ME mechanisms in action are: MHD causing upward convection in the electrolyte, MHD decreasing the diffusion layer, negative MR from spin‐channels, and POF on spin‐polarized active sites. The Pt sheet shows a negative effect and so the possible dominating ME mechanisms are: downward convection in MHD, inward Kelvin force on O_2_, pMR from the Hall effect, and pMR from spin mixing. Although, the very weak magnetization of Pt makes Kelvin force an unlikely candidate due to the extremely small magnetic gradient.

**Figure 3:**
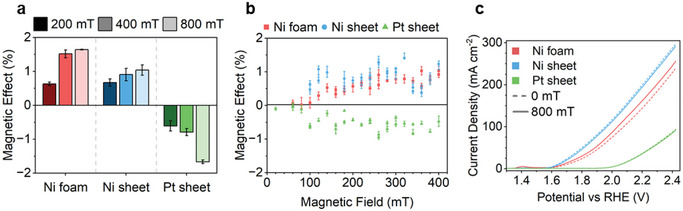
a) Percentage magnetic effect as quantified from PMCA measurements at 200, 400, and 800 mT. b) Percentage magnetic effect as quantified from PMCA measurements from 0 to 400 mT in 20 mT intervals. c) LSV curves obtained for Ni foam, Ni sheet, and Pt sheet under 0 and 800 mT fields.

PMCA experiments show an increasing magnetic field strength causes an increase in effect magnitude for all electrodes. With pulses of 200, 400, and 800 mT applied to the system, the Ni electrodes show an increase in current density upon application of a magnetic field, while a decrease in current density is observed for the Pt electrodes (**Figure** **3**a). The 0 to 400 mT region was probed further, increasing the field in 20 mT intervals (Figure 3b; Figure S4, Supporting Information). To rule out the impact of current densities on the magnetic effects, potential differences of 1.26, 1.21, and 1.7 V versus RHE were applied for Ni foam, Ni sheet, and Pt sheet, respectively, to achieve similar current densities of 120–140 mA cm^−2^. The positive and negative effects are repeatedly observed for Ni and Pt (Figure S4, Supporting Information). A general positive correlation between magnetic field strength and effect magnitude can be observed, particularly for Ni foam and Pt sheet. However, it is not clear whether the trend is linear or plateaus (Figure 3b). It should be noted that in Figure S4 (Supporting Information), current densities can be seen to slowly decay over these 4000 s experiments. This depletion in activity is a normal observation for OER systems regardless of magnetic field use – as seen in Figure S5 (Supporting Information). It can result from the lowering KOH electrolyte concentration as the OH^−^ ions are used up in the OER, or from an increasingly thick oxidized layer on the electrode's surface which decreases its conductivity.^[^ ^35^ ^]^ Over short‐term experiments, the magnetic pulses clearly do not induce any significant chemical changes to the electrode surfaces because the pre‐field current densities are regenerated post‐field – as can be observed in the example PMCAs for Ni foam in the four orientations (Figure S6, Supporting Information).

Linear sweep voltammetry (LSV) measurements (Figure 3c) show small changes on the application of the 800 mT fields: the change is positive for Ni foam, but negligible for Ni sheet and Pt sheet. The electrochemical impedance spectroscopy (EIS) data (Figure S7, Supporting Information) shows a small increase in charge transfer resistance when an 800 mT field is applied, for all electrodes. No significant changes are identified in the electrochemically active surface area (ECSA) of each electrode on the application of a magnetic field (Figure S8, Supporting Information).

Interestingly, the LSVs demonstrate a superior electrochemical performance for Ni sheet compared to Ni foam which does not align with the higher ECSA of Ni foam. This may relate to a trace metal impurity in the Ni sheet, indicated by the broad nature of the Ni^2+/3+^ anodic peak at 1.42 V versus RHE in cyclic voltammetry (CV) analysis (Figure S9, Supporting Information).^[^ ^36^ ^]^ Although, it should be noted that a Ni purity of more than 99.9% has been confirmed for both materials in further probing by SEM‐EDS and XPS (Figures S10–S17, Supporting Information), where only Ni, O, and C could be identified.^[^ ^37^, ^38^ ^]^ This trace impurity in the Ni sheet is not expected to influence the forthcoming discussion on magnetic effects and mechanisms which, in the metallic systems, is centered on the electrode magnetization.

#### 2.1.3 Mechanism Deconvolution Using Orientation

**Figure 4:**
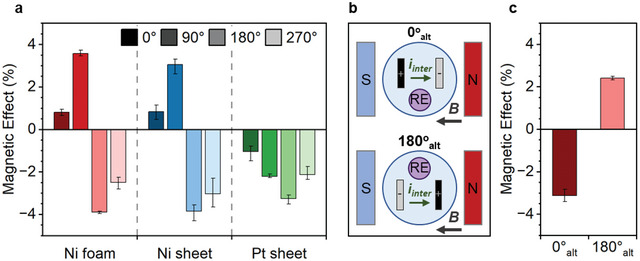
a) Percentage magnetic effect from PMCA measurements for different electrodes at 800 mT as a function of orientation (0°, 90°, 180°, and 270°). b) Schematic representation of the two alternative orientations (0° and 180°), where the WE is black, CE is grey and RE is purple. c) Percentage magnetic effect for Ni foam in the alternative orientations, where the RE positions are switched relative to the original orientations.

To further assess the ME mechanisms interacting and dominating in each system, the reactor was rotated into four positions (as illustrated previously on Figure 1b) – 0°, 90°, 180°, and 270° – and the effect of an 800 mT field was studied via PMCA measurements (**Figure** **4**a). Changing the orientation primarily affects the direction and magnitude of Lorentz force acting on *i_intra_* and *i_inter_*. The magnetic effects in all three electrode systems display significant orientation‐dependence, indicating that Lorentz force is a major contributor to the ME mechanisms. MHD was observed for all experiments and the directions of convection in each orientation are described in Figure S18 (Supporting Information). A supporting information video has also been provided of an example reaction setup in a single‐walled reactor at the 0° orientation, which clearly shows MHD occurring in the *x–y* plane.

Following is an in‐depth logical analysis of the orientation results, identifying the specific ME mechanisms in action. To ease understanding, the Ni and Pt data are discussed separately and are further broken down into parallel (0° and 180°) and perpendicular (90° and 270°) orientations – the parallel and perpendicular labels refer to the relative magnetic field and *i_inter_* vectors. The dominant effects for each system are summarized in **Table** **1**.

**Table 1:** Summary of the primary [1] and secondary [2] ME mechanisms dominating the Ni and Pt systems at each orientation. [a] and [b] indicate possible mechanisms behind the observed effect which have not been experimentally separated in this work. [+] and [−] symbols indicate whether the mechanism enhances or impedes OER performance.

Electrode	Overall	0°	90°	180°	270°
Nickel	[**1**] Lorentz force on *i_inter_* (MHD) [**2a**] Lorentz force on *i_intra_* (Hall) [**2b**] Kelvin force on O_2_	[**1+**] Lower RE‐WE resistance [**2a ‐**] Hall pMR [**2b −**] Kelvin force on O_2_	[**1+**] Upward convection	[**1 −**] Higher RE‐WE resistance [**2a −**] Hall pMR [**2b −**] Kelvin force on O_2_	[**1 −**] Downward convection
Platinum	[**1a**] Lorentz force on *i_intra_* (Hall) [**1b**] Spin‐polarization pMR	[**1 −**] pMR (Hall or spin) [**2+**] Lower RE‐WE resistance	[**1−**] pMR (Hall or spin)	[**1 −**] pMR (Hall or spin) [**2 −**] Higher RE‐WE resistance	[**1 −**] pMR (Hall or spin)

The Ni foam and Ni sheet electrodes exhibit the same pattern of orientation‐dependence, suggesting the ME effects are linked to surface magnetization and/or Ni composition (Figure 4a). The strong orientation‐dependence indicates that the ME mechanisms likely originate from Lorentz force, rather than spin polarization. If POF or nMR are dominant ME mechanisms, the magnetic effect would be positive in all orientations. Anisotropic MR could contribute as this relies on spin‐polarized charge carriers and orientation; however, the magnetic effect would likely be similar within the parallel and within perpendicular orientations.^[^ ^23^ ^]^ Additionally, MHD effects on the diffusion layer can be ruled out as a dominating mechanism; as this mechanism would be expected to cause positive magnetic effects for both perpendicular orientations, where vertical Lorentz force occurs.

The perpendicular interaction between *i_inter_* and *B* majorly contributes to the magnetic effect. For the Ni systems, the magnetic effect flips from positive at 90° (+3.6% for foam, +3.1% for sheet) to negative at 270° (−2.5% for foam, −3% for sheet). This result is in line with that of the similar perpendicular system reported by Lin et al. where photographic proof of a vertical Lorentz force effect on bubble release is shown for plate‐shaped electrodes.^[^ ^5^ ^]^ As described in the Introduction, an upward force direction promotes bubble release which causes a positive effect, and a downward force inhibits bubble release which causes a negative effect. In this work, vertical convection was visually observed in the perpendicular systems (Figure S18, Supporting Information).

In the parallel Ni systems, the magnetic effect flips from positive at 0° (+0.8%), to negative at 180° (−3.9%). As the *i_inter_* is parallel to the magnetic field, 0° and 180° are apparently equivalent systems with respect to the Lorentz force‐induced MHD effects, which makes the vastly different effects very surprising. Other works – which mainly focus on *i_inter_* – have reported changes between parallel orientations despite the systems being equivalent with respect to Lorentz force; however, no plausible explanations have been offered.^[^ ^16^, ^27^, ^34^ ^]^ In this work, the position of the RE is the only obvious change between the 0° and 180° setups (Figure 1b).

**Figure 5:**
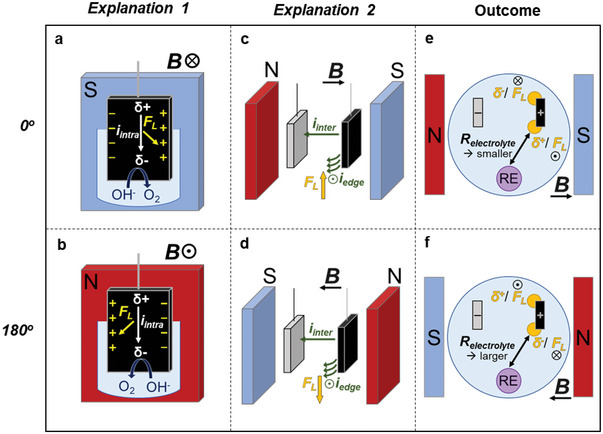
Schematic representations of parallel systems. Lorentz force affects vertical *i*_intra_, causing horizontal electrode polarization for a) 0° and b) 180°. Lorentz force created from *i*_inter_ at the electrode edges causing vertical MHD for c) 0° and d) 180°. The resulting Ohmic resistance decreasing at e) 0° and increasing at f) 180°.

Considering the RE position, two possible explanations have been developed. These center on the idea of an Ohmic drop between the RE and WE, which has been affected by unevenness in the horizontal *x–y* plane of the WE. When the Ohmic drop increases, the actual potential applied to the WE decreases, and vice versa. The application of an 800 mT field is proposed to cause Ohmic drop to decrease for 0° and increase for 180°, creating a pseudo magnetic effect (**Figure** **5**).

*Explanation 1*: horizontal charge polarization of the WE and Ohmic drop (Figure 5a,b). The vertical *i_intra_* field intersects the magnetic field orthogonally. As described by the Hall effect, the Lorentz force acts to bend charge carrier flow to one side which polarizes the electrode – in the WE, the majority carriers are of positive charge. The *δ*+ side would exhibit higher OER performance and would attract more OH^−^ ions in the electrolyte; the opposite would occur on the *δ*− side. The relative position of the RE to the *δ*+ or *δ*− side could, respectively, decrease or increase Ohmic drop via electrolyte concentration. The direction of horizontal convection observed during measurements aligns with the proposed direction of Lorentz force on *i_intra_* (Figure S18, Supporting Information).

*Explanation 2*: vertical Lorentz force at electrode edges and Ohmic drop (Figure 5c,d). The *i_inter_* field bends at the edges of the WE, creating opportunities for non‐parallel *B* and *i_inter_* interactions. Vertical Lorentz force can, therefore, result in opposite directions on either side of the electrode. The subsequent effects on bubble release could be similar to those occurring in the perpendicular systems. At 180°, downward Lorentz force restricting bubble release would occur close to the RE. The higher bubble presence would increase the WE‐RE electrolyte resistance and therefore, increase Ohmic drop. Thus, causing the apparent negative magnetic effect. The opposite would occur at 0° where the RE sits closer to the upward Lorentz force. Koza et al. report a similar observation of uneven electrolyte flow around plastic spheres under a magnetic field.^[^ ^19^ ^]^

To corroborate the Ohmic drop logic behind these explanations, additional experiments were performed for 0° and 180° WE positions using Ni foam. The relative position of the RE was flipped so that the Ohmic drop would increase for 0° and decrease for 180° (Figure 5e,f). As expected, these altered orientations indeed display a negative magnetic effect for 0°_alt_ and a positive effect for 180°_alt_.

Due to the known MHD dominance in the perpendicular Ni systems, the dominant pseudo effect in the parallel Ni systems is likely described by *Explanation 2*.

The imbalance in effect magnitudes at 0° and 180° suggests a secondary negative ME mechanism is present in the Ni systems. Building on *Explanation 1*, this could be pMR from the Hall effect resulting from Lorentz force on *i_intra_*. Alternatively, this could be a consequence of Kelvin force restricting O_2_ release or pMR from spin‐mixing; although, the latter mechanism is ruled out by the MR data presented later which shows nMR properties. A secondary negative effect is unlikely to stem from *Explanation 2*, where the effects of upward and downward convection at each edge should cancel out.

For the Pt sheet, the magnetic effects remain negative throughout the orientations but show some changes in magnitude (Figure 4a). The most likely ME mechanism dominating Pt is positive MR created from spin mixing or from the Hall effect. Vertical Lorentz force seems to be ineffective in the perpendicular orientations (90° and 270°), which both show a negative effect of −2.2%. Therefore, *i_inter_* is not a significant factor in the ME mechanisms for Pt. The different magnitudes shown for the effects at 0° (−1%) and 180° (−3.3%) results from the relative RE position. As Lorentz force from *i_inter_* is ineffective, the pseudo effect is likely caused by electrode polarization from Lorentz force on *i_intra_*, as described in *Explanation 1*.

The Pt electrode's dependency of ME mechanisms on *i*_intra_ is a different observation to that reported in the literature. Lin et al. report the magnetic effect of a Pt plate system to flip from positive to negative when the reactor is rotated from 90° to 270°.^[^ ^5^ ^]^ The weaker influence on *i_inter_*‐derived convection in the Pt sheet system reported in this work may be due to differing parameters, such as larger inter‐electrode distance (15 mm) compared to the literature system (10 mm), electrode shape, magnetic field strength, and potential difference.

The Ni and Pt systems exhibit different dependencies on *i_inter_* in the effective ME mechanisms. This likely stems from the significantly stronger magnetization of ferromagnetic Ni, leading to a high magnetic flux density about the electrode. Thus, the local electrolyte environment is under a stronger influence from Lorentz force and the resulting effect of MHD on OER performance is much larger.

Spin‐originated MR analysis was conducted on the electrode materials in the catalysis‐equivalent parallel and perpendicular orientations (Figure S19, Supporting Information). Any Hall contributions have been removed by symmetrizing the data. Both Ni materials display isotropic nMR of similar magnitude (≈−0.7% at 800 mT) which evidently is not a major contributor to the catalytic magnetic effects. This differs from a literature report by Zhang et al. where the nMR of a Ni foam core, which sits beneath a NiFe‐LDH/Co_3_O_4_ surface, is shown to improve OER activity.^[^ ^39^ ^]^ The authors conclude that nMR dominates their system whereas the Ni electrodes reported in this work are dominated by MHD; this difference in effect domination presumably stems from the electrode surface composition.

A small anisotropic pMR of ≈+0.01% at 800 mT was measured for the Pt material. The MR measurements represent the material's bulk properties; however, the surface will likely have more electron scattering events due to the larger abundance of lattice defects. It is therefore possible that the effective pMR in surface‐sensitive catalytic reactions is much larger than what these measurements indicate.^[^ ^40^, ^41^ ^]^

In conclusion, the strong magnetization of the Ni foam and Ni sheet electrodes creates a strong ME dependence on electrolyte convection – affecting mass transfer – which is derived from Lorentz force on the *i_inter_*. On the other hand, the behavior of the Pt sheet is controlled by the intra‐electrode charge resistance – affecting electron transfer with adsorbates – which is derived from Lorentz force on *i_intra_* from spin‐pMR. The pseudo‐RE electrode effect can be created from Lorentz force on *i_inter_* and *i_intra_* and thus, is present in all the metallic systems.

#### 2.1.4 Surface Controlled MHD

Building on the conclusion of the *i_inter_*‐derived Lorentz force dominating the Ni electrodes, further insights can be drawn by comparing the magnetization and geometry of Ni foam and Ni sheet.

The magnetization data in Figure 2a,b show that while Ni foam and Ni sheet have similar mass magnetizations, Ni sheet exhibits a much higher electrode‐volume magnetization. If the absolute magnetization of the electrode that is, the total number of polarized electrons in the entire electrode – is the primary determinant of the magnetic effect, Ni sheet would be expected to exhibit a significantly larger magnetic effect than Ni foam. However, the observed magnetic effect magnitudes for Ni foam and Ni sheet are similar. Suggesting that magnetization about the electrode surface plays a more critical role than bulk magnetization in determining the magnetic effect.

Developing this idea, while the ECSA of Ni foam is approximately five times larger than that of Ni sheet (Figure S8, Supporting Information), the magnetic effect magnitude does not scale proportionally. This indicates that the magnetic effect is not directly proportional to the total surface magnetization at an atomic scale. Instead, it is concluded that the overall plate‐like geometry of the electrode, and its associated surface magnetization, exert the most significant influence on MHD‐induced magnetic effects.

### 2.2 Spin‐Polarization Domination in Co_3_O_4_ and the Role of BaFe_12_O_19_ as a Magnetic Co‐Catalyst

The work extends to a powder‐based Co_3_O_4_/BaFe_12_O_19_ series which demonstrates dominant spin‐polarization mechanisms. The electrodes were fabricated by drop‐casting the powders onto carbon paper (see Experimental Section). Co_3_O_4_ has been chosen as a model OER electrocatalyst due to its optimal binding energies toward adsorbed oxygen intermediates.^[^ ^42^ ^]^ The OER reaction on Co_3_O_4_ tends to follow the adsorbate evolution mechanism where there are four electron transfer steps: i) OH^−^ adsorbs to the Co^2+^ or Co^3+^ site to give *OH, ii) deprotonation occurs to give *O, iii) the intermediate is then attacked by a secondary OH^−^ to give *OOH, and iv) final deprotonation occurs to give molecular O_2_ and the Co‐active site is regenerated.^[^ ^43^, ^44^ ^]^ BaFe_12_O_19_ has been chosen as a magnetic co‐catalyst due to its strong ferromagnetic properties. The co‐catalyst enhanced Co_3_O_4_ magnetic effects through increased flux density.

#### 2.2.1 Electrode Characterization

**Figure 6:**
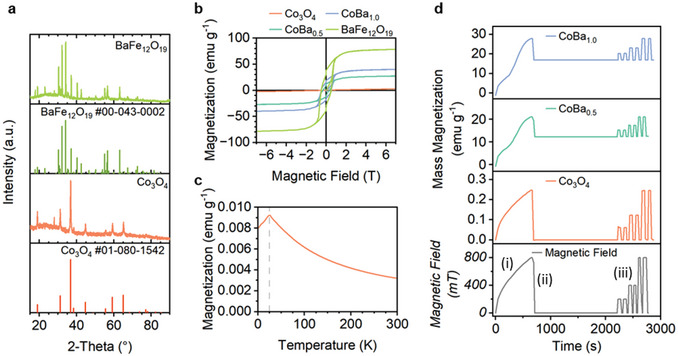
a) XRD data obtained for Co_3_O_4_ and BaFe_12_O_19._ b) Mass magnetization versus magnetic field plots for Co_3_O_4_, CoBa_0.5_, CoBa_1_, and BaFe_12_O_19_. The peak at 28° in both spectra is caused by silicone grease used in the sample preparation (Figure S22, Supporting Information). c) Mass magnetization versus temperature from 0 to 300 K for Co_3_O_4_. d) Plots of mass magnetization versus time under a sequence of magnetic field strengths: i) slow field ramp up to 800 mT, ii) field removal to 0 mT, iii) ≈50 s field pulses between 0 mT and 200, 400, and 800 mT. These plots demonstrate the magnetic remanence of each electrode, where the ≈50 s pulses mimic the PMCA experiments.

Co_3_O_4_ was synthesized using a hydrothermal method. A pure crystal structure is confirmed by XRD (**Figure** **6**a) and rod morphology is shown in TEM images (Figure S20, Supporting Information). BaFe_12_O_19_ was synthesized via a sol–gel method. The crystal structure is confirmed by XRD (Figure 6a) and a plate morphology is shown in TEM images (Figure S21, Supporting Information).

Magnetometry analysis of Co_3_O_4_ (Figure 6b) shows a linear relationship between magnetization and magnetic field strength, reaching 0.54 emu g^−1^ at 800 mT and 2.76 emu g^−1^ at 7000 mT. Analysis of magnetization versus temperature (Figure 6c) confirms an antiferromagnetic property which is in agreement with the literature.^[^ ^45^ ^]^ Magnetometry analysis of BaFe_12_O_19_ (Figure 6b) reveals strong ferromagnetism with a *M_800_* of 54.3 emu g^−1^, *M_s_* of 78.4 emu g^−1^, coercivity of 490.3 mT, and remanence of 38.1 emu g^−1^. Mixtures of Co_3_O_4_ and BaFe_12_O_19_ were prepared in weight ratios of 1:0.5 and 1:1, denoted herein as CoBa_0.5_ and CoBa_1_. The mass magnetization increases with BaFe_12_O_19_ content (Figure 6b), with the *M*_s_ of CoBa_0.5_ and CoBa_1_ reaching 27.3 and 40.1 emu g^−1^, respectively.

The retentivity of each sample was measured (Figure 6d) under a slow 800 mT pulse and under ≈50 s pulses which mimic PMCA conditions. The Co_3_O_4_ relaxes fully and immediately after the external field is removed. The magnetization of the BaFe_12_O_19_ composites partially relax to 61% for CoBa_0.5_ and 58% for CoBa_1_ after the initial slow application of the field to 800 mT due to the high remanence of BaFe_12_O_19_. After 25 mins, quick PMCA‐mimic pulses were applied; with each pulse, magnetization relaxes down to the base‐level retentivity of ≈60%. In the PMCA catalysis experiments, the electrodes have all been previously exposed to an 800 mT field. Therefore, it is likely that for completely unmagnetized electrodes, the reported magnetic effects for the BaFe_12_O_19_ composites would be larger than are reported in this work. To avoid this problem, superparamagnetic single crystals of BaFe_12_O_19_ may be used in future studies.

#### 2.2.2 OER in a Magnetic Field

Initial experiments on magnetic effects were conducted in the 0° orientation. The addition of BaFe_12_O_19_ is shown to enhance the magnitude of the positive magnetic effect for Co_3_O_4_. This increase in Co_3_O_4_ performance may result from spin‐polarized active sites favoring POF, nMR through spin‐channels, or MHD from Lorentz force. Literature reports on Co_3_O_4_ in similar systems tend to focus on POF, and one report discusses the impact of nMR.^[^ ^4^, ^26^, ^29^ ^]^ Unfortunately, the MR of the Co_3_O_4_ synthesized in this work could not be measured; the sample was observed to have an extremely large resistance (>100 MΩ) which went beyond the capabilities of the physical property measurement system (PPMS).

**Figure 7:**
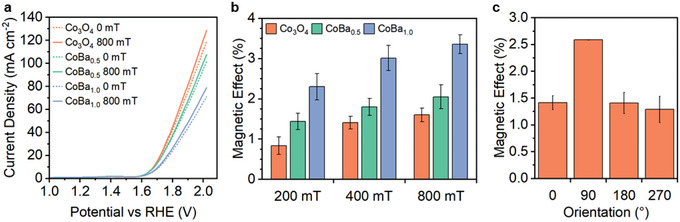
a) LSV curves obtained for Co_3_O_4_, CoBa_0.5_, and CoBa_1_ under 0 and 800 mT fields. b) Percentage magnetic effect as quantified from PMCA measurements at 200, 400, and 800 mT. c) Percentage magnetic effect from PMCA measurements at 200 mT as a function of orientation (0°, 90°, 180°, and 270°) for Co_3_O_4_.

Electrochemical characterization by LSV shows Co_3_O_4_ to have the highest performance in the series (**Figure** **7**a). Whereas, BaFe_12_O_19_ is essentially inactive, reaching only 1.63 mA cm^−2^ at 2 V versus RHE (Figure S23, Supporting Information). Therefore, BaFe_12_O_19_ in the mixture systems can only act as a magnetic co‐catalyst. Interestingly, the catalytic activity of the mixtures decreases with increasing BaFe_12_O_19_ content (Figure 7; Figure S24, Supporting Information). This is likely a result of the increase in powder loaded onto the carbon paper: 2 mg cm^−2^ on Co_3_O_4_, 3 mg cm^−2^ on CoBa_0.5_, and 4 mg cm^−2^ on CoBa_1_. As is well documented in the literature, the additional powder acts as a barrier between the active Co_3_O_4_ particles and the carbon paper electrode‐core; which increases the distance for electron transfer and hence, decreases OER performance.^[^ ^46^, ^47^, ^48^ ^]^ The ECSA analysis also indicates that BaFe_12_O_19_ may partially block Co_3_O_4_ active sites (Figure S25, Supporting Information), although the physical mixing will still allow for diffusion pathways to most active sites.

Under an applied magnetic field, the performance of all electrodes increases. Positive changes can be observed in the LSV measurements (Figure 7a), although the PMCA measurements offer a more accurate approach to magnetic effect analysis, as discussed above. Interestingly, in the PMCA data, a positive trend emerges for magnetic effect versus BaFe_12_O_19_ content. Co_3_O_4_, CoBa_0.5_, and CoBa_1_ show increasing magnetic effects at 800 mT of +1.60%, +2.05%, and +3.36%, respectively (Figure 7b). EIS analysis shows an 800 mT magnetic field decreases charge transfer resistance for all three systems (Figure S26, Supporting Information).

The role of BaFe_12_O_19_ is to increase the magnetic flux density about the Co_3_O_4_ catalyst. This increases its magnetization and therefore, its receptivity to ME enhancement.

The possibility of electrochemically‐induced Fe‐migration from BaFe_12_O_19_ into the Co_3_O_4_ surface has also been considered as a possible cause of ME enhancement. XRD, CV, and SEM‐EDS analyses were conducted to evaluate this point. The XRD patterns of CoBa_1_ before and after electrocatalysis show no changes (Figure S27, Supporting Information). The Co^2+/3+^ anodic peak at ≈1.4 V during CV conditioning increases in intensity and does not shift for Co_3_O_4_, CoBa_0.5_, and CoBa_1_ (Figure S28, Supporting Information). If Fe‐migration was occurring here, the peak intensity would decrease as the Co‐active sites become blocked and would also shift to higher energies.^[^ ^49^, ^50^ ^]^ Finally, SEM‐EDS analysis of Co_3_O_4_ and CoBa_1_ before and after electrocatalysis (Figures S29–S35, Supporting Information) show no evidence of Fe‐migration. The Co_3_O_4_ rods are entirely composed of Co and O in every sample. Considering the very low detection limit of SEM‐EDS (≈0.1 at%), and the XRD and CV analyses, it can be concluded that if Fe‐incorporation into the Co_3_O_4_ has occurred, it is negligible. Therefore, this type of event is unlikely to create ME enhancement of Co_3_O_4_ in the mixture systems.^[^ ^37^ ^]^

Interestingly, the carbon paper electrode base itself shows a negative magnetic effect which, similar to the Pt electrode, may result from pMR (Figures S36 and S37, Supporting Information). Whilst the earlier study on Ni foam indicated that ME magnitude is primarily determined by the electrode surface, a previous study by Zhang et al. reported magnetic effects in an MR‐dominated system being dependent on the MR of the electrode core. Therefore, the carbon paper may be partially counteracting the positive effects of Co_3_O_4_.

#### 2.2.3 Mechanism Deconvolution Using Orientation

To evaluate the impact of Lorentz force, PMCA measurements were conducted at different orientations – 0°, 90°, 180°, and 270° – for the Co_3_O_4_ system (Figure 7c). The magnetic effect has been found to be largely independent of orientation, except for the 90° orientation where a secondary ME mechanism appears to contribute. Similar to the Ni systems, this is due to an upward Lorentz force promoting bubble release from the surface. A coupled negative effect may also be expected at the opposite perpendicular orientation of 270°. This discrepancy has previously been seen in a Co_3_O_4_/Ni foam system, reported by Li et al., and it may demonstrate the negative effect of downward Lorentz force canceling out the positive effect of bubble dispersion.^[^ ^34^ ^]^ Additionally, MHD effects on the diffusion layer can be ruled out as a dominating mechanism, due to the similar magnitude of magnetic effects at 0°, 180°, and 270°. This mechanism would be expected to cause positive magnetic effects for both perpendicular orientations, where vertical Lorentz force occurs, but would have minimal effect in the parallel orientations.

The consistent positive effect at each orientation rules out Lorentz force as the primary source of dominant ME mechanisms, narrowing the candidates down to POF and nMR which arise from spin‐polarization. Unlike the metallic electrodes in the previous series, where the effects are strongly dependent on Lorentz force, the Co_3_O_4_ systems are powder‐based and so are less likely to act as one macro‐plate unit. This hinders the synergic interaction of the multiple *i_intra_* and *i_inter_* Lorentz force events occurring across the electrode. nMR could not be directly evaluated in this work due to the Co_3_O_4_ sample having an unmeasurably large resistance, but the mechanism is possible according to the literature.^[^ ^4^ ^]^ The possibility of POF is assessed by density functional theory (DFT) calculations in the following section.

#### 2.2.4 POF Compatibility Using DFT

**Figure 8:**
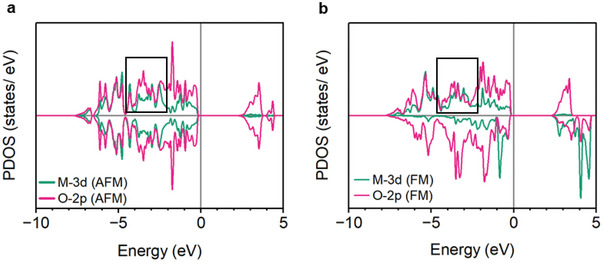
PDOS plots for Co^2+^ and O^2−^ from tetrahedral sites in Co_3_O_4_ in a) antiferromagnetic and b) ferromagnetic states.

To assess the possibility of the POF mechanism on the Co_3_O_4_ surface, DFT calculations were conducted. The projected density of states (PDOS) of Co_3_O_4_ was studied, focusing on Co^2+^ (M‐3d) tetrahedral sites. PDOS data for the antiferromagnetic (AFM) and ferromagnetic (FM) states are shown in **Figure** **8**. The unit cell parameters measured from XRD data for Co_3_O_4_ are *a* = 8.09 Å with a cubic Fd‐3m space group. The DFT calculations yield *a* = 8.11 Å and the same space group, which are in excellent agreement with the experiments. It should be noted that the complete FM state (saturation of Co_3_O_4_ magnetization) is impossible to reach in experimental conditions where fields up to 800 mT were used, and saturation magnetization was not reached by 7000 mT in the magnetometry analysis (Figure 6; Figure S38, Supporting Information). PDOS is used to study the extent of M‐3d‐to‐O‐2p hybridization and therefore, the strength of this covalent bond. As seen in the PDOS plots (Figure 8), there is a better overlap between M 3d and O 2p lines in the *E* range of [−4, −2] eV for the FM state (highlighted region) which indicates stronger 3d‐2p hybridization (Figure S39, Supporting Information). This facilitates ferromagnetic exchange which restricts the spin of the lattice oxygen, thus promoting spin‐selective spin transfer from the O adsorbates to the lattice O. These results are in agreement with a previous study by Ren et al. where the structure of CoFe_2_O_4_ with and without spin alignment was modeled.^[^ ^26^ ^]^ These computational results strengthen the argument that spin‐polarized POF is a key ME mechanism leading to improved OER performance.

## 3 Conclusion 

This work provides a systematic framework for understanding and analyzing ME mechanisms in the OER, particularly by using reactor orientation to isolate Lorentz force effects and logical comparisons of different electrode systems under identical conditions. Such findings highlight the interplay of electrode composition, geometry, and magnetism in determining ME effects.

For the metallic Ni and Pt electrodes, Lorentz force has been shown to govern the magnetic effects. The Ni electrodes are majorly influenced by Lorentz force on the *i_inter_* which affects the mass transport of the reaction, indicated by the systems’ strong orientation‐dependences. Comparisons between Ni foam and Ni sheet reveal that surface magnetization, rather than bulk magnetization, primarily governs the magnetic effect from MHD. In contrast, the lower magnetic flux density of Pt weakens any local magnetic effects on *i_inter_* and so, Pt is dominated by pMR from Lorentz force on *i_intra_* and from spin‐polarization. These magnetic effects negatively impact the performance of Pt, by slowing the electron transfer steps in the OER reaction.

Interestingly, this study also identifies a “pseudo” effect arising from the relative RE position in parallel systems which are dominated by Lorentz force; emphasizing the importance of careful experimental setup design. The unevenness across the horizontal *x–y* plane of the WE could be a consequence of the Hall effect on *i_intra_* and/or local vertical MHD on the *i_inter_* at the edges of the WE plate. These findings should be applied in all future magneto‐electrocatalytic experiments using a RE.

The Co_3_O_4_ powder‐based system is dominated by spin‐polarization mechanisms. The persistent positive effect likely arises from both nMR and POF which target the electron transfer steps of the OER reaction. Ferromagnetic BaFe_12_O_19_ has successfully been used as a magnetic co‐catalyst, creating a larger flux density about Co_3_O_4_ to enhance the magnetic effect magnitude. However, to prevent the drop in absolute catalytic performance, further work is required to fine‐tune the use of magnetic co‐catalysts.

Ultimately, the goal of mechanistic studies is to find a route to optimized the use of magnets in OER. Catalyst and reactor design should be used as tools to probe which properties favor positive mechanisms, and which favor negative mechanisms. The system design can then be optimized to maximize magnetic enhancement. The work reported here demonstrates the first step in this process. Using orientation and two separate electrode designs, different Lorentz force and spin‐polarization mechanisms have been isolated. From here, the properties needed for mechanistic control can begin to be understood. Excitingly, the Co_3_O_4_ powder system at 90° combines Lorentz force and spin‐polarization enhancement, setting an example for how to get the best out of both.

## 4 Future Perspectives

To deepen understanding of how experimental design features link to different ME mechanisms, future research strategies should pursue the key topics: i) electrode composition, ii) electrode shape, iii) magnetic co‐catalysts, iv) field direction and strength, and v) reactor configuration.

In particular, metallic powders deposited on carbon paper should be investigated. This should reveal whether the observed ME preferences in metallic plate and powder‐based systems in this work, arise from the atomic composition of the electrode surface or from broader morphological and structural factors. Additionally, exploring the effects of surface versus bulk magnetization, such as by coating Ni electrodes with other catalysts and varying the coating thickness, could offer valuable insights.

Reference electrodes are an important feature of many laboratory‐scale electrochemical reactions. Untangling the pseudo mechanisms may assist future researchers in conducting more accurate assessments of real ME mechanisms. This could be approached by modeling/probing the horizontal charge distribution of the electrode or by imaging bubble flow at the edges.

As magneto‐electrocatalytic water‐splitting moves closer to scale‐up, researchers should begin to consider the catalyst stability. Particularly, by looking into possible catalyst degradation through attraction to the external magnet and how different ME mechanisms affect catalyst lifetime.

The compatibility of common commercial electrolytic cells with a magnetic field should also be a key point of study. This may initiate a branch of study for engineers, to produce compatible cells which facilitate enhancement mechanisms.

Through greater control of the MHD effect, there could be possible applications for facilitating gas separation in current industrial electrolyzers. The parallel orientations create circular convection in the *x–y* plane of the reactor. This convection pushes H_2_ and O_2_ in opposite directions. Specialized reactors could be built to capitalize on this effect, capturing the gases in separate regions. However, further work is initially required to understand the actual composition of gas bubbles in different regions during MHD.

Finally, specific technoeconomic assessments on the industrial and economic feasibility of magneto‐electrocatalytic water‐splitting is essential. These are needed to guide researchers toward the best routes for moving effective systems toward scale up. The reports should consider factors such as the choice between a permanent magnet or an electromagnet, equipment costs, running costs, magnet lifetime, and safety considerations.

Through progress and success in these areas, magneto‐electrocatalytic water‐splitting could become a realistic option for sustainable and efficient green H_2_ production.

## 5 Experimental Section

### Catalyst Synthesis

The co_3_O_4_ catalyst was synthesized via a hydrothermal method.^[^ ^51^ ^]^ First, Co(NO_3_)_2_·6H_2_O (873 mg, 3 mmol), NH_4_F (74 mg, 2 mmol), and urea (300 mg, 5 mmol) were dissolved in deionized water (80 mL). The solution was then transferred to a 100 mL Teflon‐lined stainless‐steel autoclave and kept in an oven for 7 h at 140 °C. The resulting solid sample was washed with deionized water and ethanol and dried in a vacuum oven at 60 °C. Afterward, the sample was calcined in air at 400 °C for 3 h with a heating rate of 2 °C min^−1^.

BaFe_12_O_19_ was synthesized via a citrate sol–gel method.^[^ ^52^ ^]^ Three solutions were made up:

*Solution A*: Ba(NO_3_)_2_ (117.6 mg, 0.45 mmol) in ultrapure water (5 mL)

*Solution B*: Fe(NO_3_)_3_·9H_2_O (2.09 g, 5.17 mmol) in ultrapure water (15 mL)

Solution C: citric acid (2.16 g, 11.2 mmol) in ultrapure water (11.2 mL)

Solutions A, B, and C were carefully mixed together at room temperature and the pH was adjusted to 7 by dropwise addition of NH_4_OH (30 wt.%). The resulting solution was heated to 90 °C with continuous stirring and maintained at this temperature until a viscous gel was formed (≈2 h). Subsequently, the gel was heated at 340 °C for 4 h to give the precursor powder. The sample was then transferred into a tube furnace and heated in air at 850 °C for 6 h with a heating rate of 5 °C min^−1^.

### Preparation of Working Electrodes—Metal Catalysts

Nickel foam, nickel sheet, and platinum sheet were cleaned ultrasonically with acetone, water, and ethanol to remove surface impurities and dried under air. Active geometric surface area (1 × 1 cm) was used for the three metal catalysts.

### Preparation of Working Electrodes—Powder Catalysts

Catalyst ink was prepared by making a suspension of the catalyst powder (2 mg of Co_3_O_4_) and Nafion 5 wt.% binder (15 µL) in ethanol (200 µL). The catalyst ink was then drop casted onto carbon paper to form a working electrode (WE) (2 × 1 cm with an active surface area of 1 × 1 cm).

The process was repeated for the Co_3_O_4_/BaFe_12_O_19_ mixture electrodes (only the amount of catalyst powder was changed); using Co_3_O_4_ (2 mg) plus BaFe_12_O_19_ (1 mg) for CoBa_0.5_, and Co_3_O_4_ (2 mg) plus BaFe_12_O_19_ (2 mg) for CoBa_1_.

### Electrochemical Measurements

All electrochemical measurements were performed using a CHI604E potentiostat in a water‐cooled reactor containing a conventional 3‐electrode system with Hg/HgO reference electrode and platinum sheet counter electrode (1 × 1 cm) in 1 m KOH electrolyte. The interelectrode distance between the WE and CE was fixed at 1.5 cm. All measured potentials were converted with respect to the reversible hydrogen electrode (RHE) using Equation (2) and are presented without *iR* correction.

(2) $$E_{\mathrm{RHE}}=E_{\mathrm{Hg}/\mathrm{HgO}}+0.059\left(\mathrm{pH}\right)+0.098$$

LSV measurements were recorded at a scan rate of 10 mV s^−1^ in the potential range of 0–1.1 V versus Hg/HgO unless otherwise stated. EIS measurements were recorded at 0.9 V versus Hg/HgO in the frequency range from 10 kHz to 0.1 Hz with an amplitude of 0.1 V.

### Magneto‐Electrochemical Experiments

An electromagnet was used to conduct electrochemical experiments under external applied magnetic fields (Figure S1, Supporting Information). The field strength was adjusted by changing the applied current of the electromagnet and calibrated using a Gauss meter. In pulsed magneto‐chronoamperometry (PMCA) experiments, the first 350 s were recorded under no magnetic field to allow stabilization of current, followed by multiple 50 s magnetic field pulses at specified field strengths.

### Characterization

Powder XRD was performed on a Bruker D8 Advance X‐ray diffractometer. CCDC #00‐004‐0850, #00‐004‐0802, #01‐080‐1542, #00‐043‐0002 contain the supplementary crystallographic data for this paper. This data can be obtained free of charge from The Cambridge Crystallographic Data Centre via www.ccdc.cam.ac.uk/data_request/cif. Magnetic property measurements were performed on a Quantum Design MPMS‐XL5 superconducting quantum interference device to give the magnetization versus field and magnetization versus time graphs. Four‐point magnetoresistance measurements were conducted on a Quantum Design PPMS system with metal electrodes cut into 5 × 1 mm rectangles. XPS analysis was performed using a Thermo NEXSA XPS fitted with a monochromated Al Kα X‐ray source (1486.7 eV). Survey scans were recorded at a pass energy of 200 eV, and high‐resolution scans recorded at a pass energy of 40 eV. SEM‐EDS data was recorded using a Zeiss Sigma 300 SEM with an Oxford Instruments Ultim Xtreme X‐ray detector for topographical and elemental imaging. TEM‐EDS data was collected on a Talos F200X G2 (accelerating voltage: 200 kV) and EDS was acquired using a Super‐X Detection System.

### DFT Calculations

All DFT calculations were performed using the Vienna Ab initio Simulation Package (VASP).^[^ ^53^ ^]^ The exchange‐correlation interactions within the Kohn–Shame scheme were described by the Perdew–Burke–Ernzerhof (PBE) functional in the framework of the generalized gradient approximation (GGA). Grimme's D3 dispersion‐correction scheme (DFT‐D3) was adopted to account for the van der Waals interactions.^[^ ^54^, ^55^ ^]^ The initial structure information of bulk Co_3_O_4_ was obtained from the XRD experiment. The Co_3_O_4_ unit cell, which includes 56 atoms, was used in the calculations. To converge to the FM and AFM states, spin‐polarized calculations were carried out, with initial guesses for the magnetic moments explicitly specified on each atom by setting the “MAGMOM” tag. All atoms were allowed to fully relax during the geometric optimization. The electronic wavefunctions were expanded in a plane‐wave basis set with a cutoff energy of 520 eV to avoid errors due to the Pulay stress.^[^ ^56^ ^]^ The energy convergence tolerance for the electronic problem was set to 10^−5 ^eV, and for the geometric optimization the tolerance for the Hellmann–Feynman forces was set to 0.01 eV Å^−1^. The Brillouin‐zone was sampled with a *Γ*‐centered homogeneous *k*‐point mesh based on the Monkhorst–Pack scheme. The *k*‐point meshes for FM and AFM Co_3_O_4_ were set to 8 × 8 × 8 according to the literature. To better simulate the 3d electronic systems, the Hubbard U corrections of 4.4 and 6.4 eV were applied for all Co^2+^ and Co^3+^ 3d electrons using the Dudarev approach to compensate for the onsite self‐interaction error, with all *U*_eff_ (*U*_eff _= *U* − *J*) parameters as in previous studies.^[^ ^57^ ^]^ The 3D spin density and electron localization function were post‐processed from the self‐consistent field calculation of VASP and visualized using VESTA software (Figure S39, Supporting Information).

## Conflict of Interest

The authors declare no conflict of interest.

## Author Contributions

A.R. and D.S. contributed equally to this work. A.R. and D.S. prepared, characterized, tested the catalysts with the help of Q.C. and M.Y. M.L. and M.S. carried out the DFT calculations. A.R. and D.S. wrote the paper with the help of Y.L. and X.P. All authors read and revised the paper. C.W. and S.C.E.T. supervised the overall project.

## Supporting information



Supporting Information

Supplemental Video 1

## Data Availability

The data that support the findings of this study are available from the corresponding author upon reasonable request.
