# Supplementary material for: Untangling the Mechanisms in Magneto‐Electrocatalytic Oxygen Evolution
Source: Small. 2025 May 2;22(12):2412852. doi: 10.1002/smll.202412852 (PMC12934389; doi:10.1002/smll.202412852)
Supplement: Supplementary file 1 — Supporting Information [file SMLL-22-2412852-s002.docx]

Supporting Information

**Untangling the Mechanisms in Magneto-Electrocatalytic Oxygen Evolution**

Amy Radford^#^, Dorottya Szalay^#^, Qiming Chen, Mengfan Ying, Mingyu Luo, Xuelei Pan, Michail Stamatakis, Yiyang Li*, Chen Wu*, Shik Chi Edman Tsang*

Figures S1 – S39 are presented in this file.


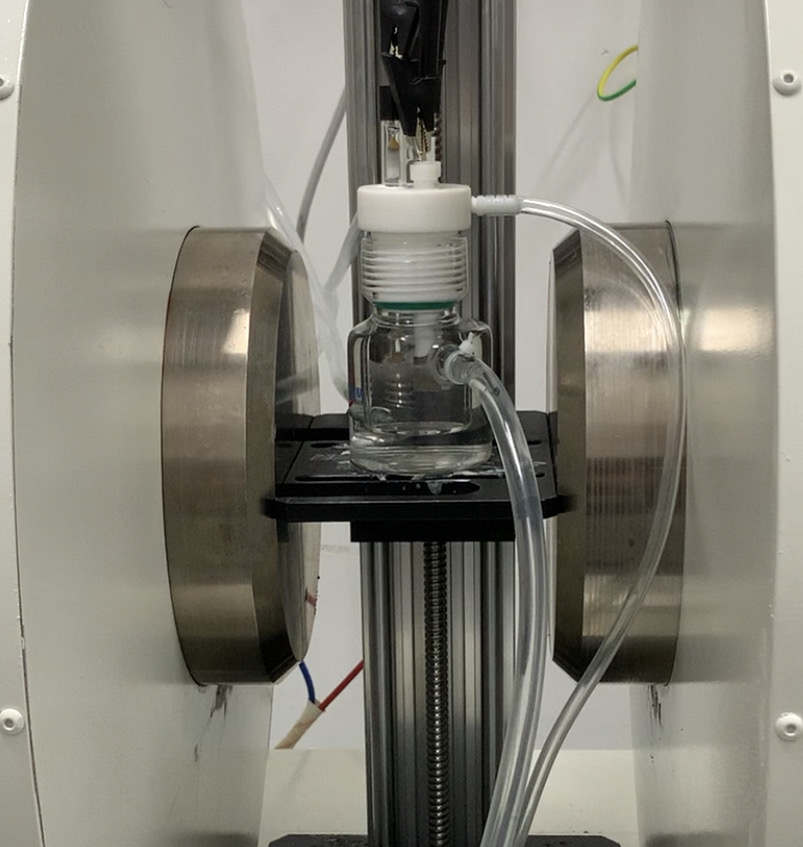


**Figure S1.** Photograph of water-cooled electrochemical reactor in electromagnet, in the 90^o^ orientation.


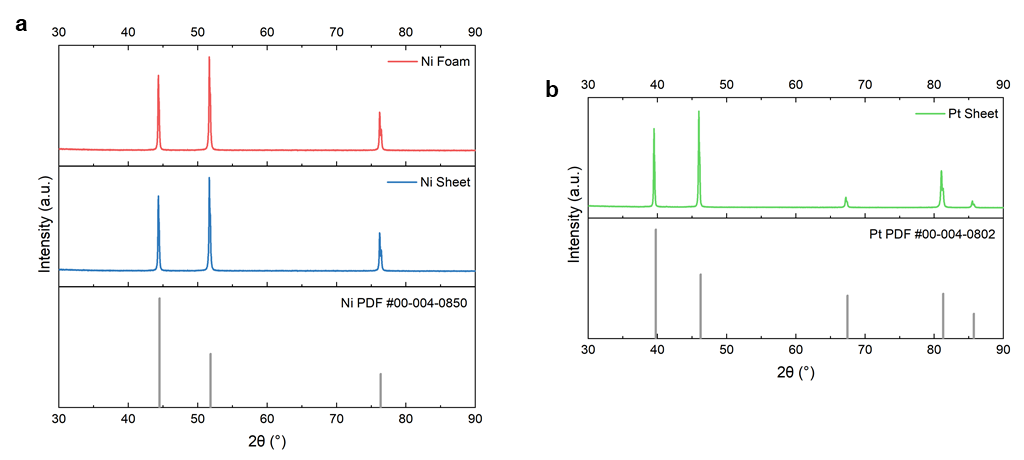


Figure S2. XRD data obtained to confirm crystal structure of commercial materials used for electrodes: *a*) Ni foam and Ni sheet, and *b*) Pt sheet.


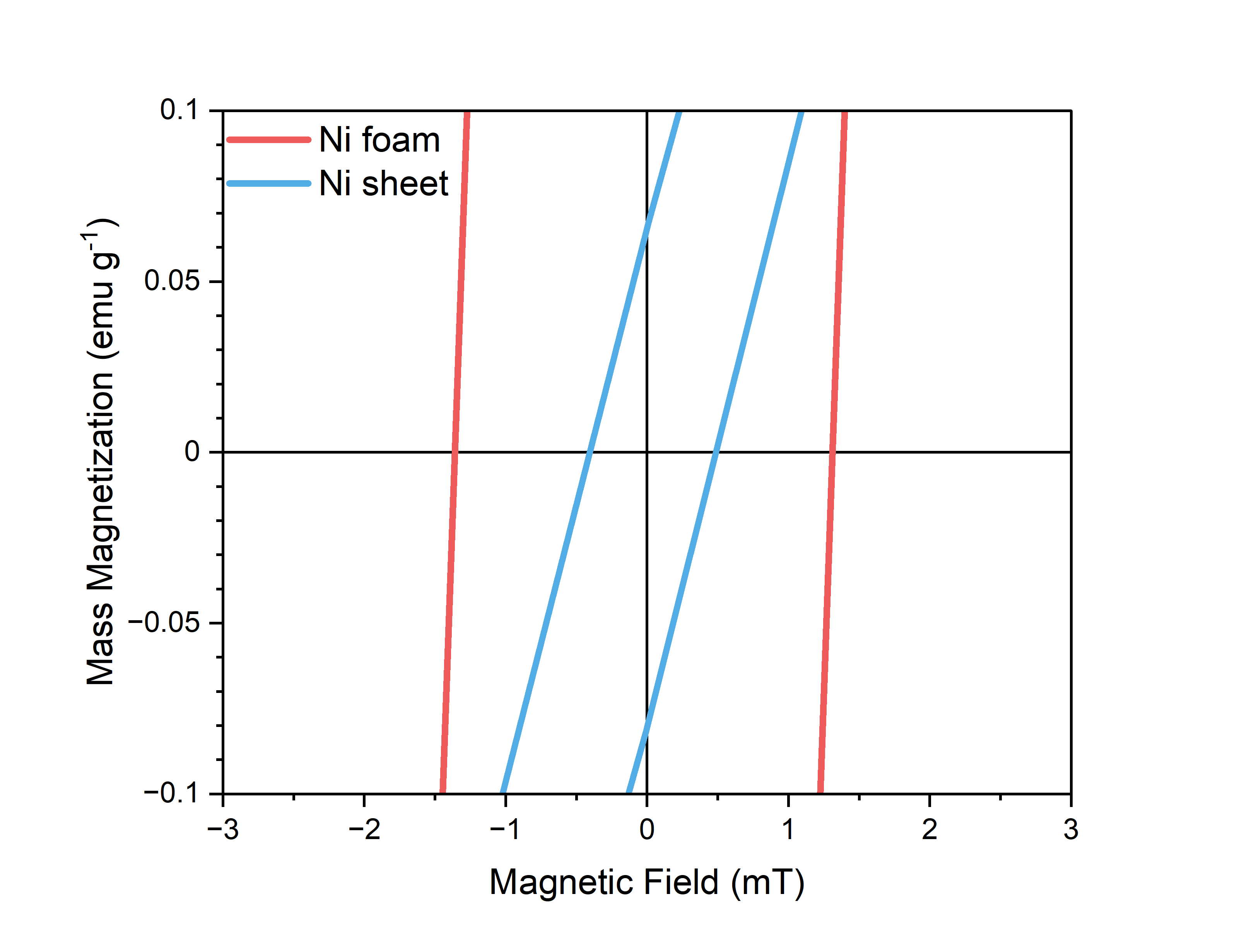


Figure S3. Coercivity of ferromagnetic Ni foam and Ni sheet.


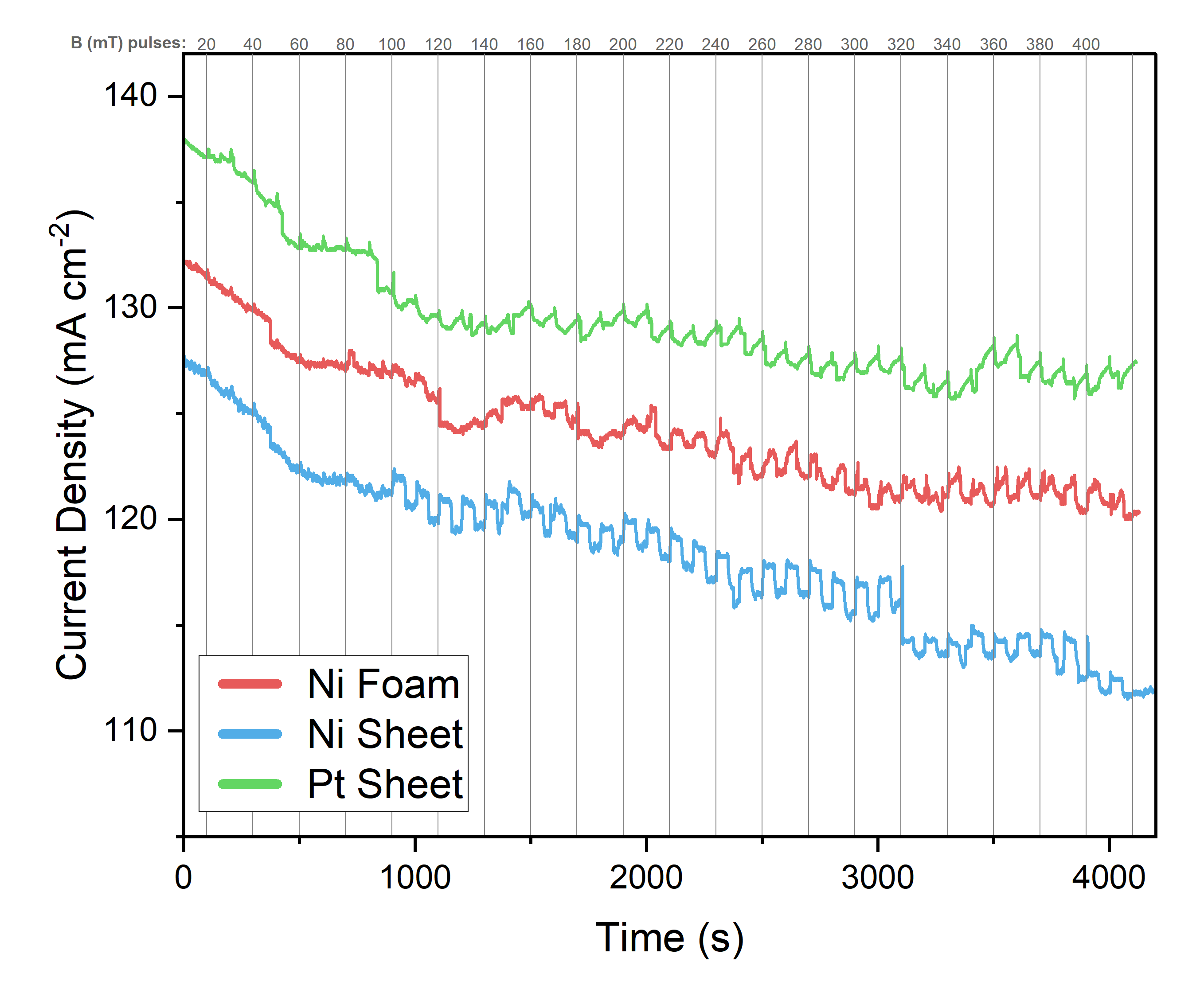
Figure S4. Plots of PMCA measurements scanning 0 to 400 mT in 20 mT intervals using the metallic electrodes. In calculation of the percentage magnetic effect, anomalous points were removed due to some fluctuations in the system which are normal over long measurement periods.


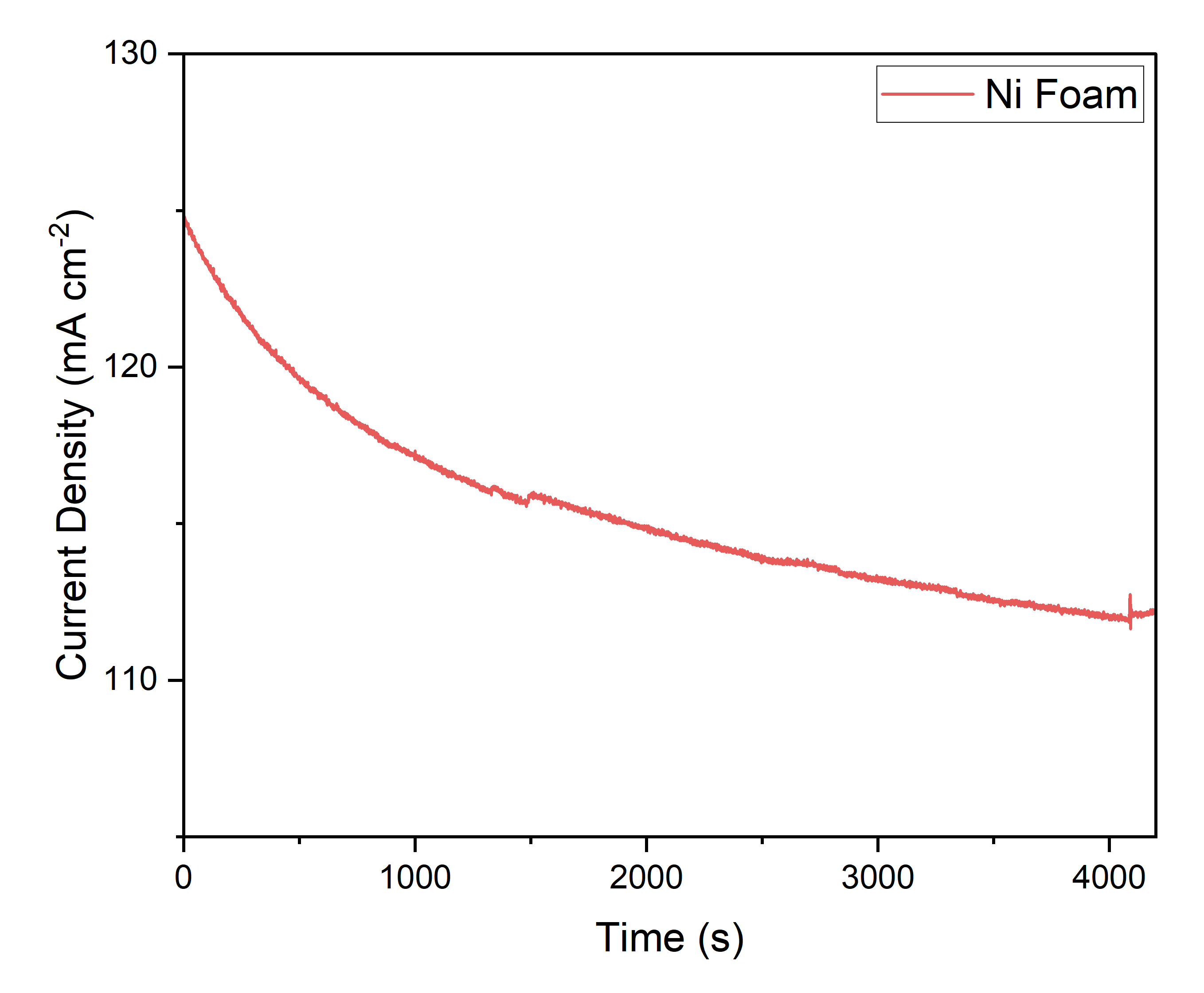


Figure S5. Plots of CA measurement using Ni foam in similar reaction conditions to the PMCA experiments in Figure S4. No magnetic field applied here. This demonstrates the natural degradation of Ni foam OER performance over time.


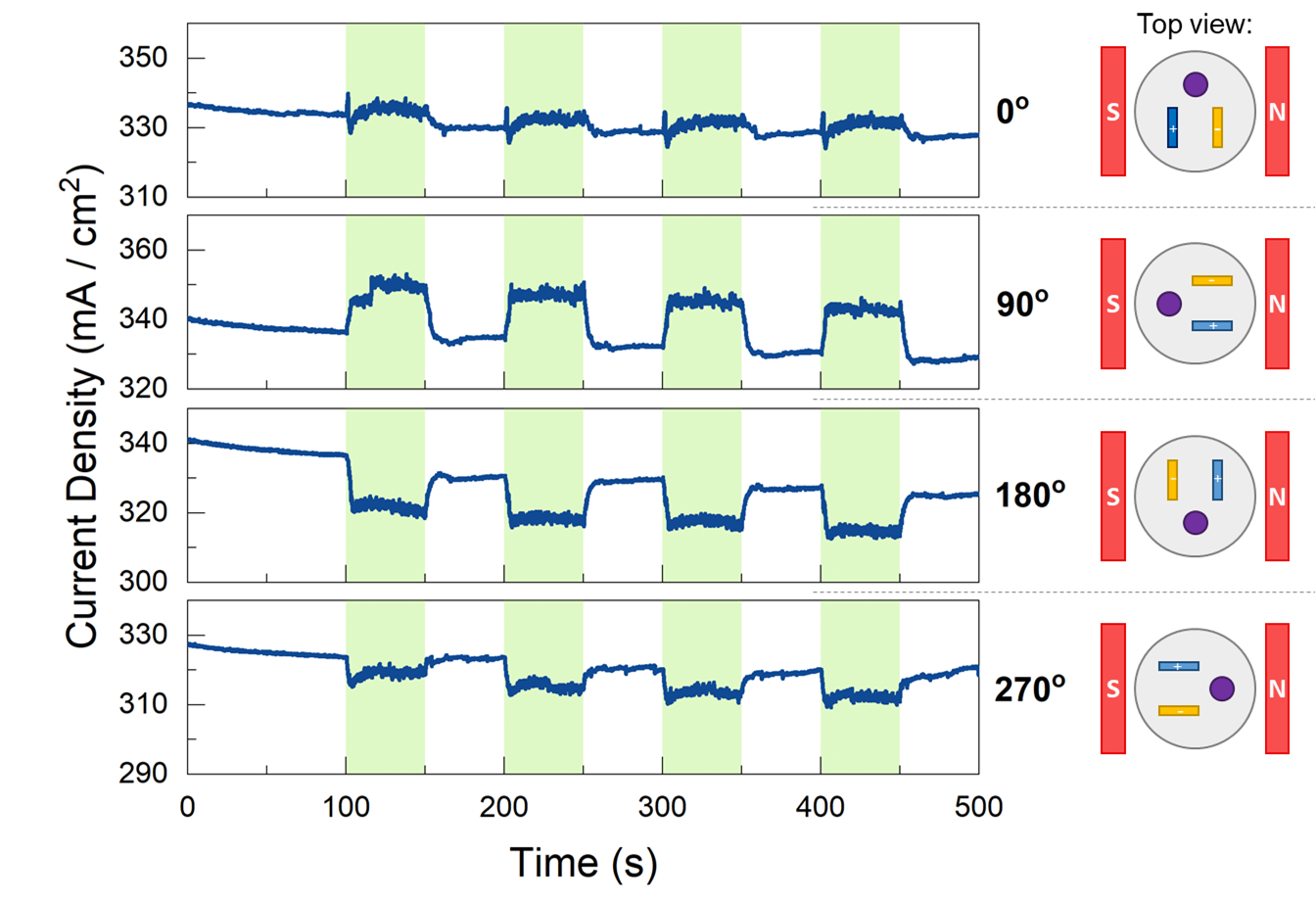


Figure S6. Plots of PMCA measurements for Ni foam in four orientations: 0^o^, 90^o^, 180^o^, and 270^o^. 800 mT pulses were applied in 50 s pulses, indicated by the green shaded regions.


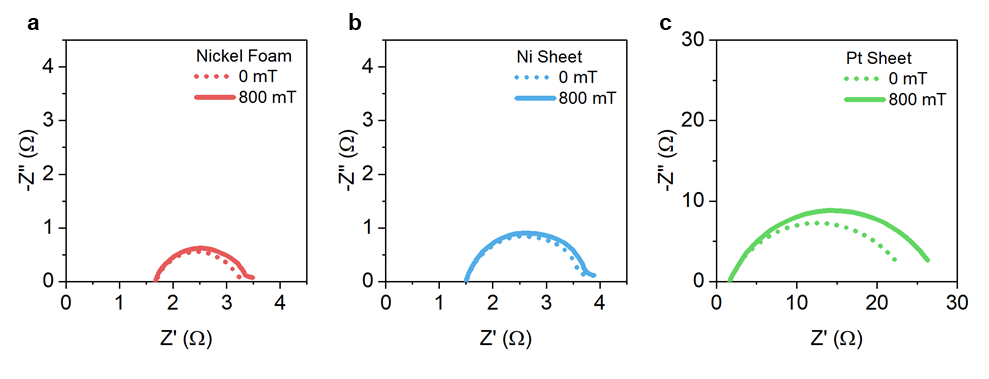


Figure S7. EIS plots taken at 1.8 V vs RHE for *a*) Ni foam, *b*) Ni sheet, and *c*) Pt sheet under 0 and 800 mT fields.


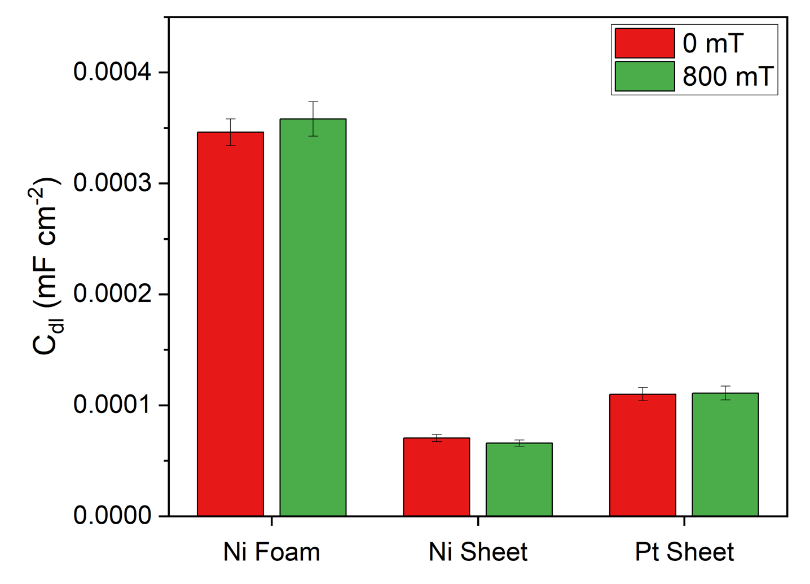


Figure S8. Double layer capacitance (directly proportional to ECSA) for Ni foam, Ni sheet, and Pt sheet under and 800 mT.


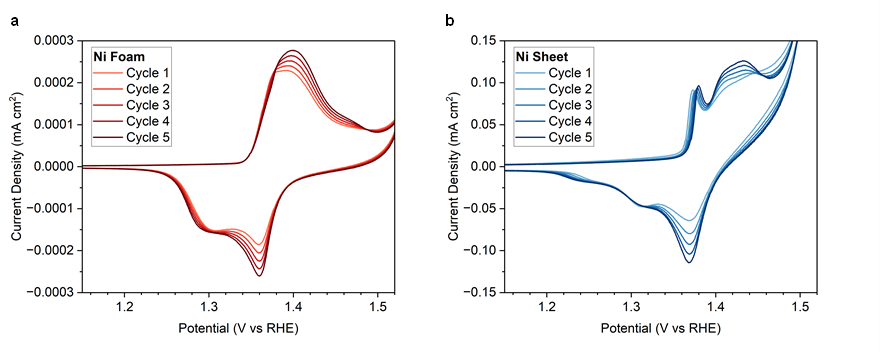


Figure S9. CV plots conditioning, cycles 1 to 5, for *a)* Ni foam and *b)* Ni sheet. Focussed on the redox region.


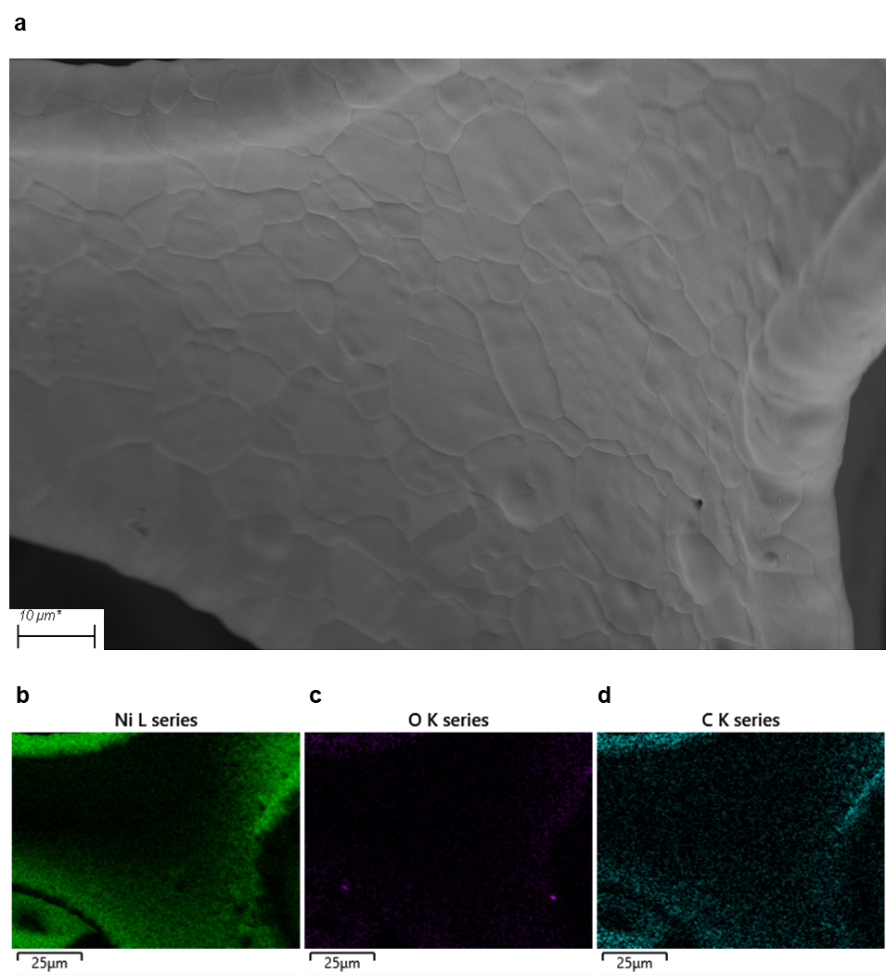


Figure S10 *a)* SEM image of Ni foam pre-OER. EDS mapping of *b)* Ni, *c)* O and *d)* C content.


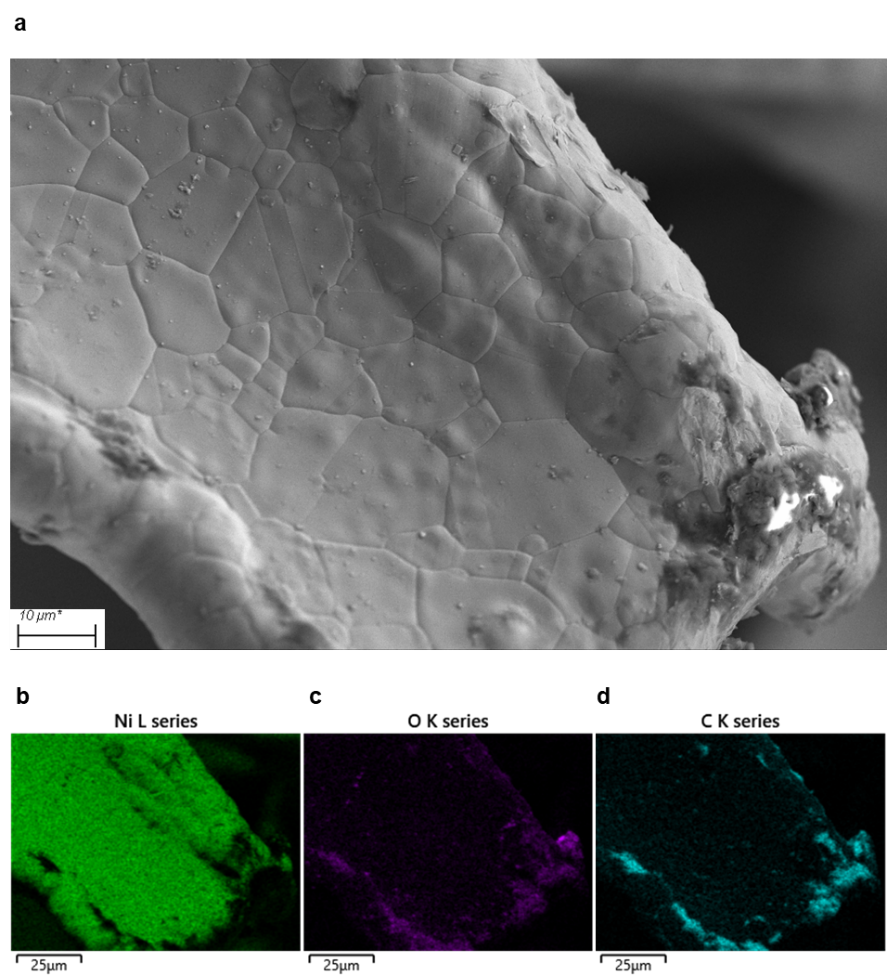


Figure S11. *a)* SEM image of Ni foam post-OER. EDS mapping of *b)* Ni, *c)* O and *d)* C content.


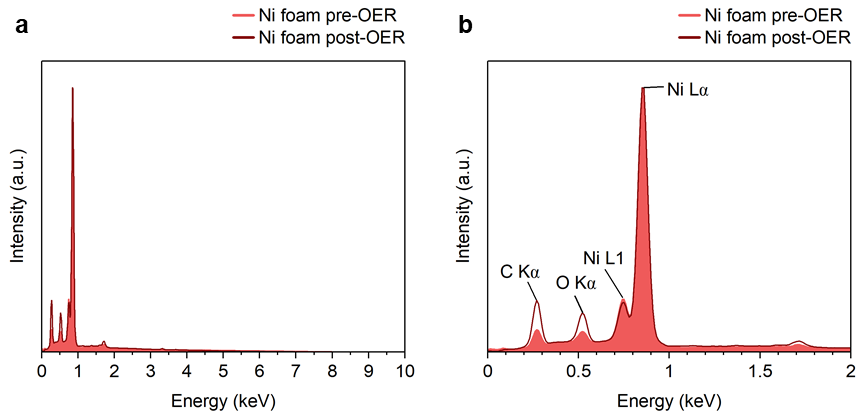


Figure S12. EDS plots of Ni foam pre- and post-OER: *a)* full scan and *b)* zoomed in on region 0-2 keV. The peak at 1.7 keV results from pulse pile-up of the Ni Lα X-ray of 0.85 keV.


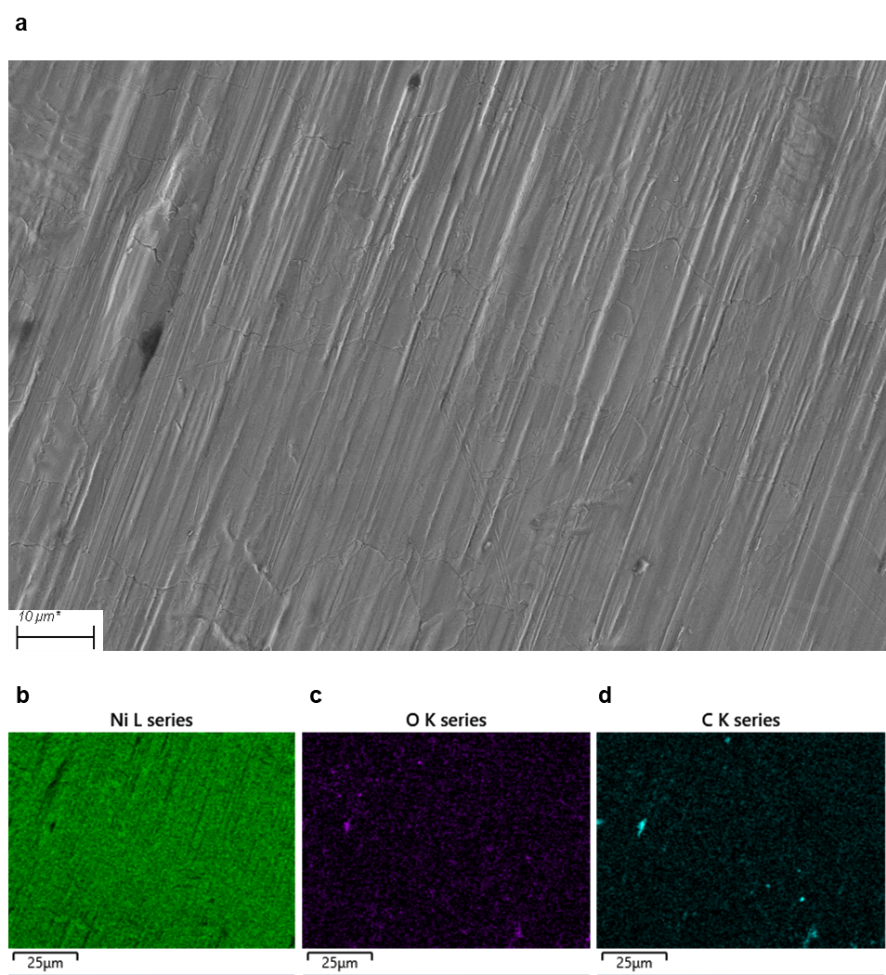


Figure S13. *a)* SEM image of Ni sheet pre-OER. EDS mapping of *b)* Ni, *c)* O and *d)* C content.


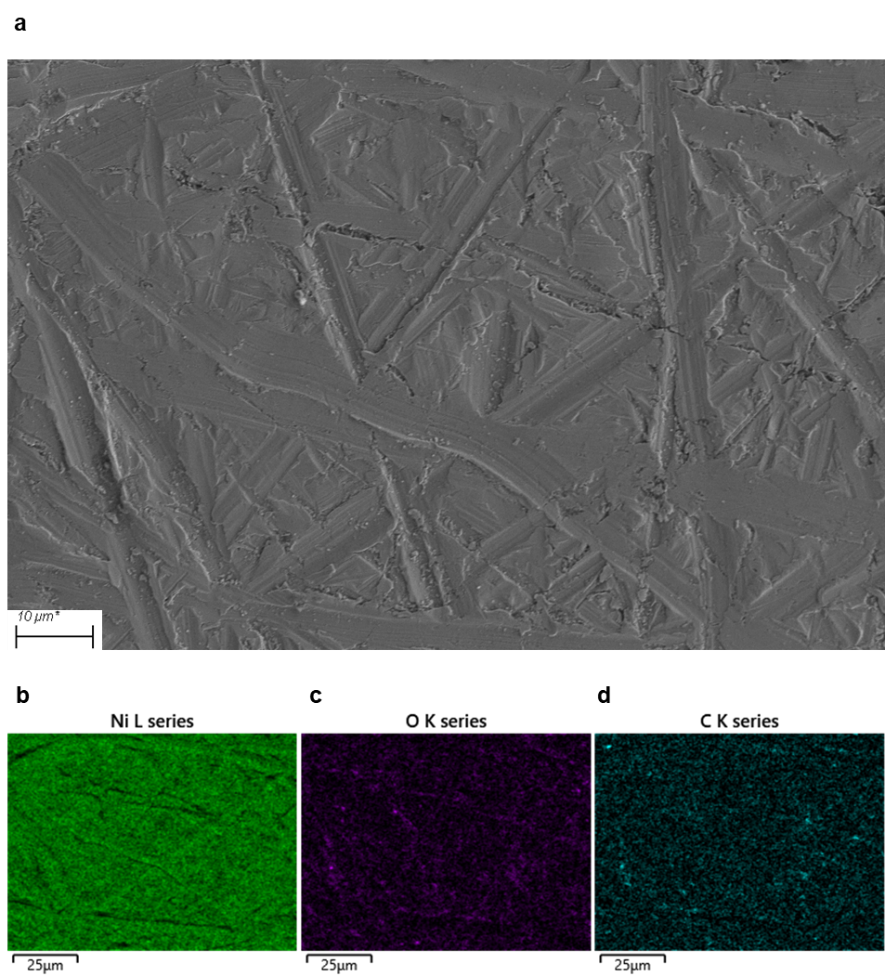


Figure S14. *a)* SEM image of Ni sheet post-OER. EDS mapping of *b)* Ni, *c)* O and *d)* C content.


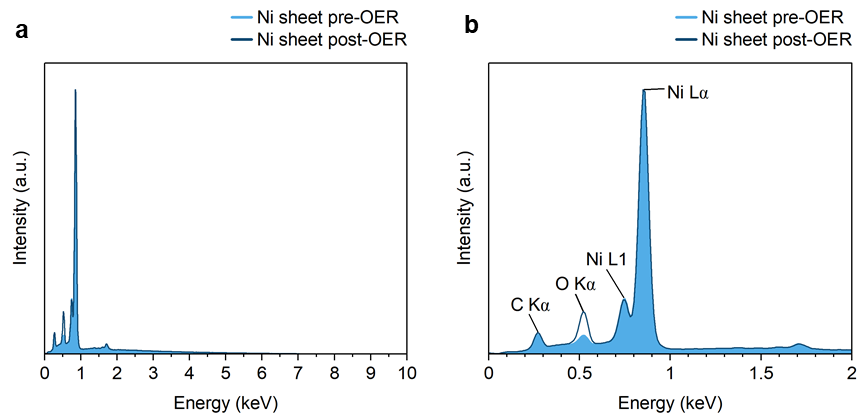


Figure S15. EDS plots of Ni sheet pre- and post-OER: *a)* full scan and *b)* zoomed in on region 0-2 keV. The peak at 1.7 keV results from pulse pile-up of the Ni Lα X-ray of 0.85 keV.


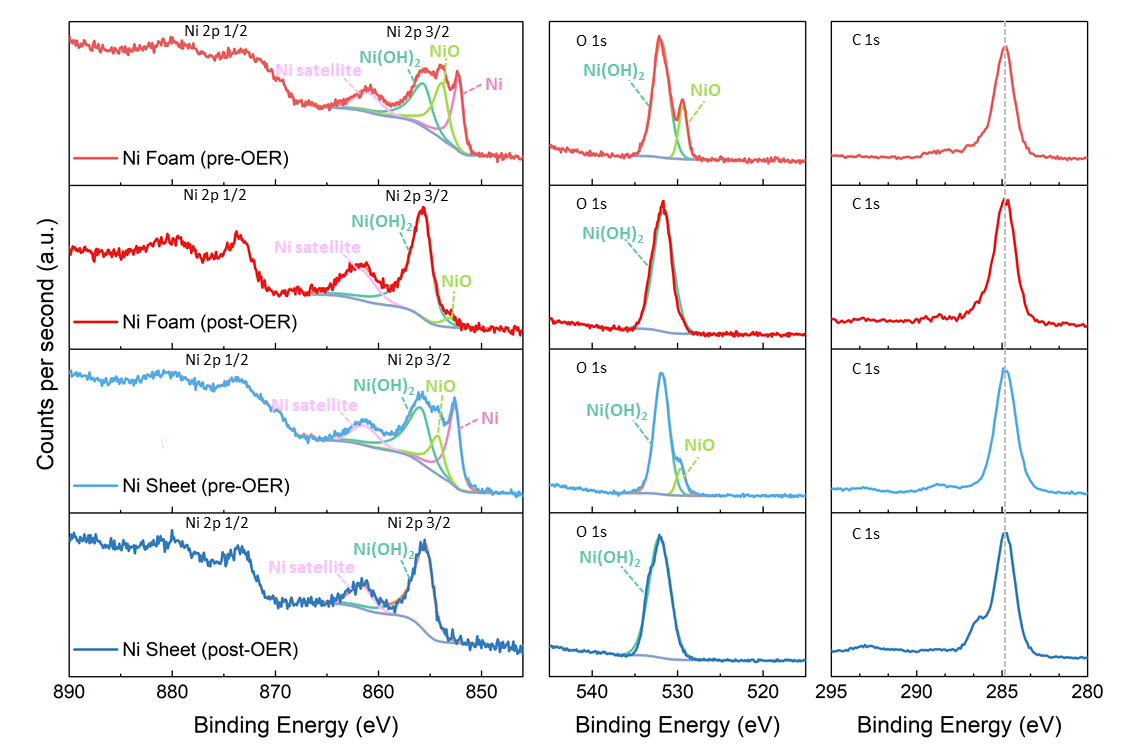


**Figure S16.** XPS spectra for Ni foam and Ni sheet before catalysis and after catalysis (2.4 V applied for 1 hr in OER). C 1s spectra show the adventitious C peaks aligned at 284.8 eV and was used for calibration of all spectra. Ni 3d spectra show Ni, NiO, and Ni(OH)_2_ species on Ni sheet and Ni foam, and show oxidation of the Ni surface to Ni(OH)_2_ after catalytic use. O 1s spectra also show NiO and Ni(OH)_2_ present in the pre-OER specimens and only Ni(OH)_2_ post-OER.


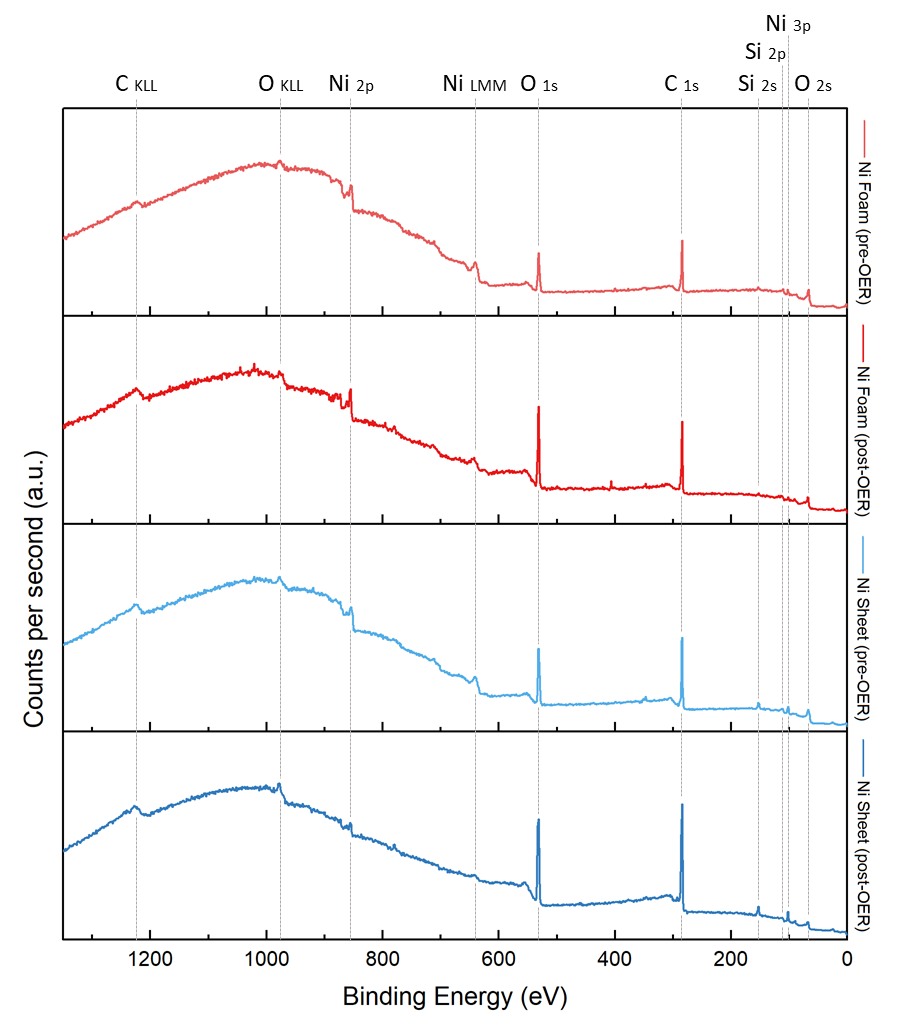


Figure S17. Wide-scan XPS spectra for Ni foam and Ni sheet before catalysis and after catalysis (2.4 V applied for 1 hr in OER). The spectra were calibrated using C 1s at 284.8 eV (Figure S16). The Si peaks are likely instrumental background.


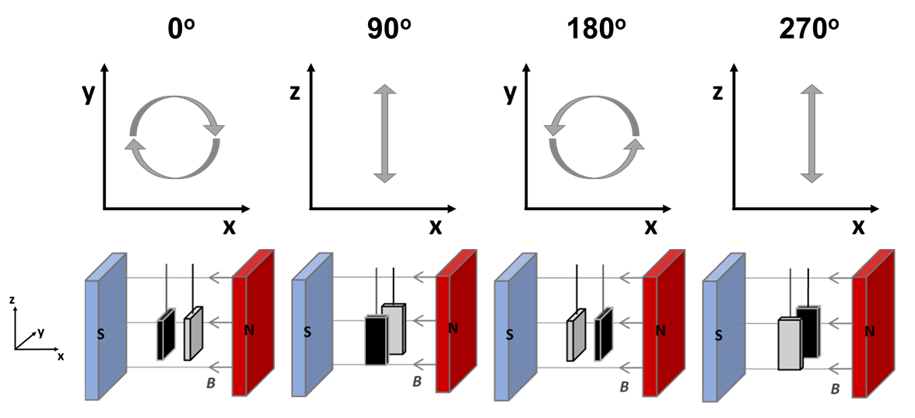


Figure S18. Schematics demonstrating electrolyte convection observed for each orientation during the magneto-electrocatalytic measurements of the metallic electrode series.


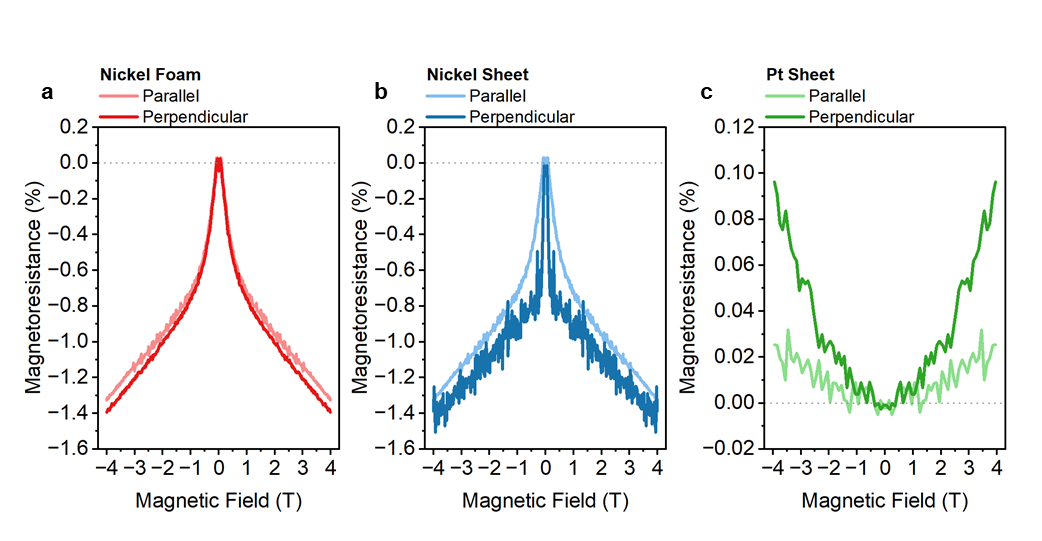


Figure S19. Magnetoresistance plots of *a*) Ni foam, *b*) Ni sheet, and *c*) Pt sheet. Where the magnetic field is perpendicular to the direction of current flow (5 mA), the parallel orientation refers to out-of-plane alignment to the electrode surface, and the perpendicular orientation refers to in-plane alignment. The data was symmetrized to remove Hall contributions.


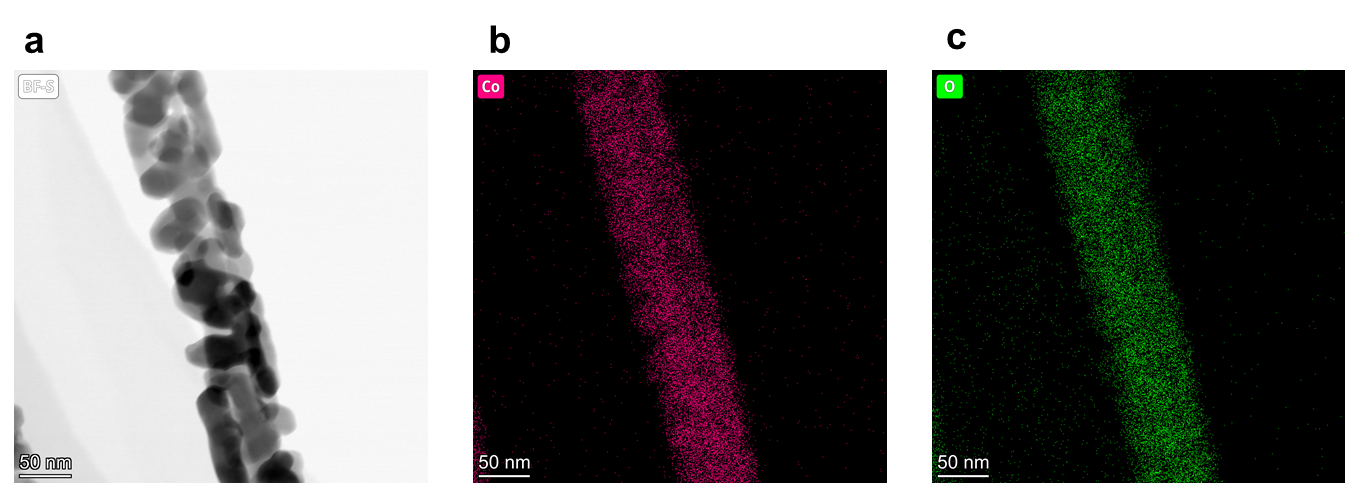


**Figure S20. a)** TEM image of Co_3_O_4_ rod. EDS mapping of **b)** Co and **c)** O content.


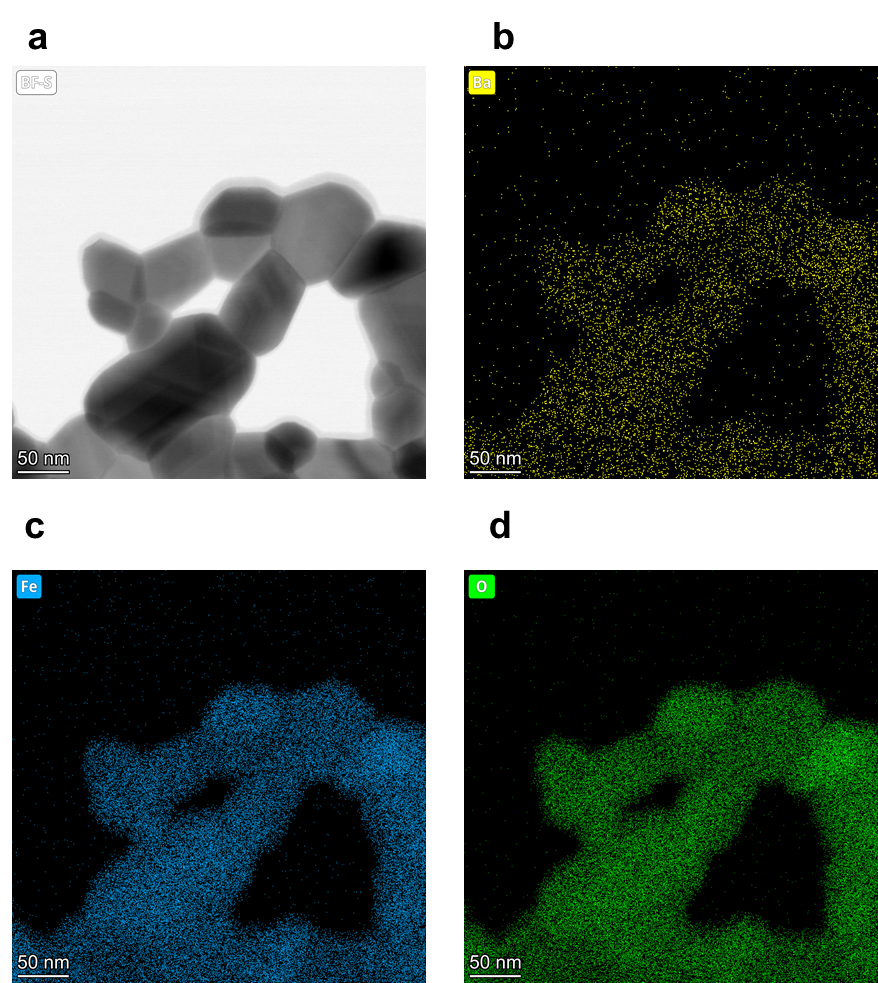


Figure S21. a) TEM image of.BaFe_12_O_19_ showing plate-like structure. EDS mapping of b) Ba c) Fe and d) O.


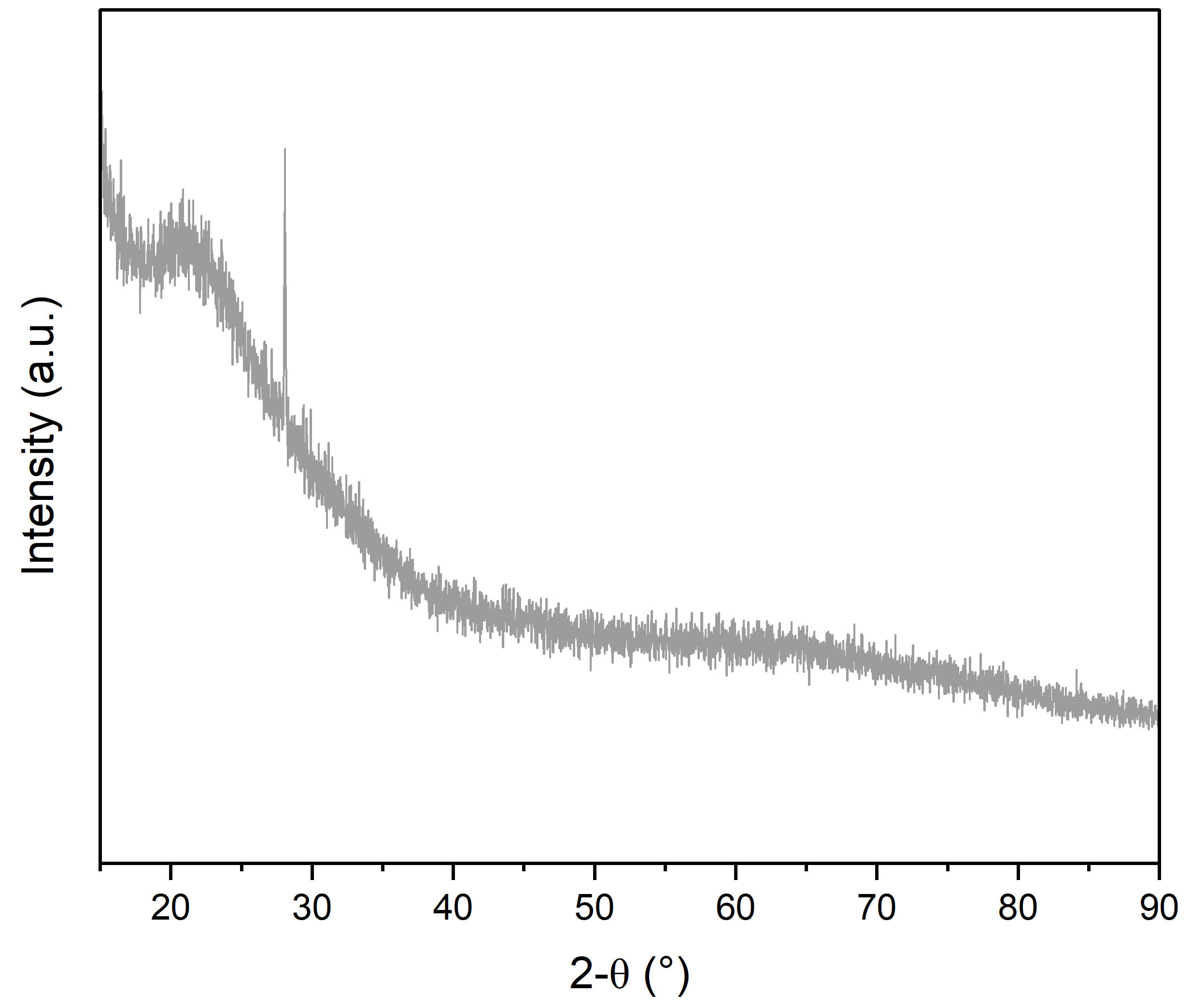
Figure S22 XRD pattern of the sample holder background, confirming that the peak at 28^o^ seen in Figure 6a is a result of the silicone grease used in sample preparation.


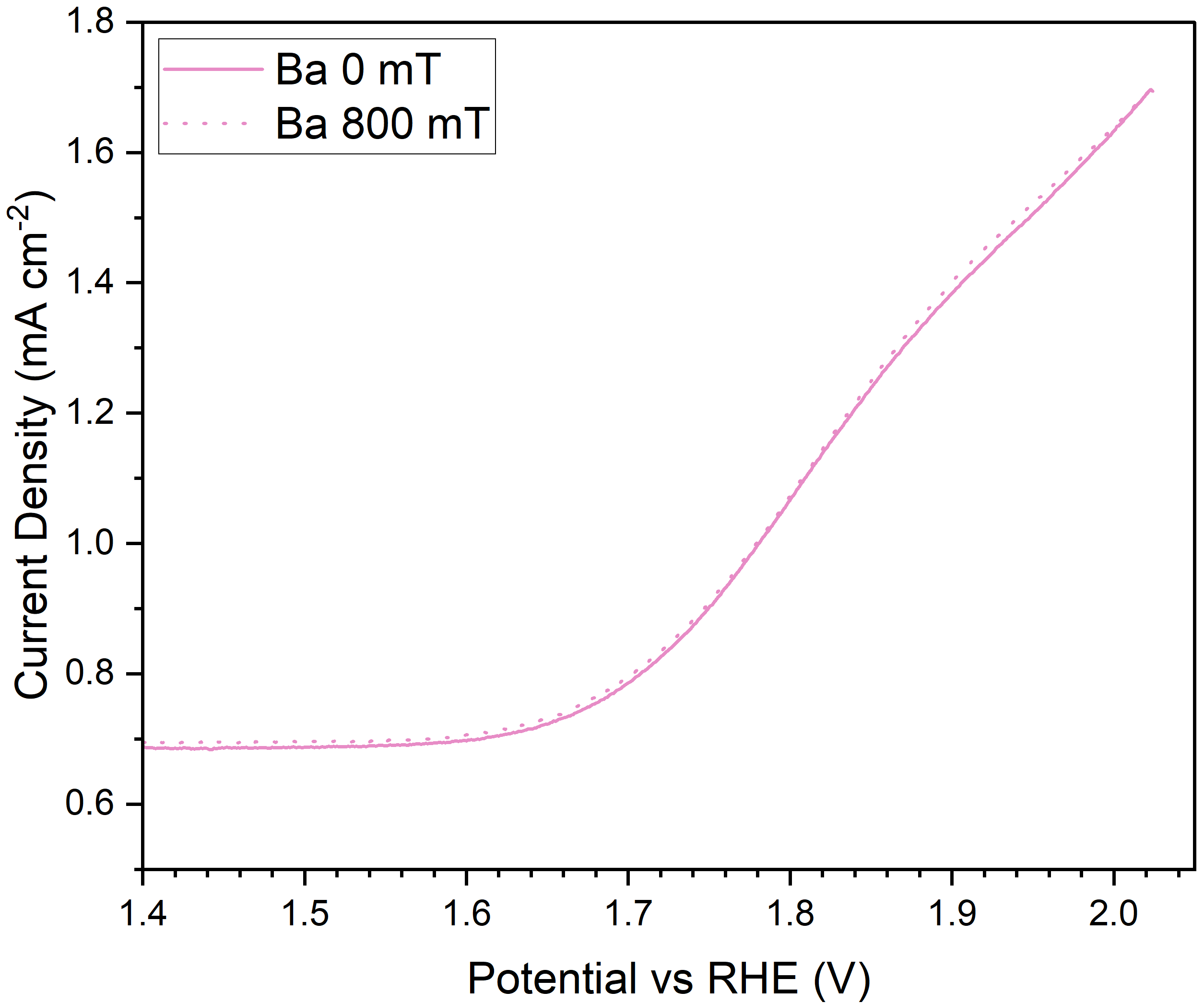


Figure S23. LSV measurement of BaFe_12_O_19_ on carbon paper.


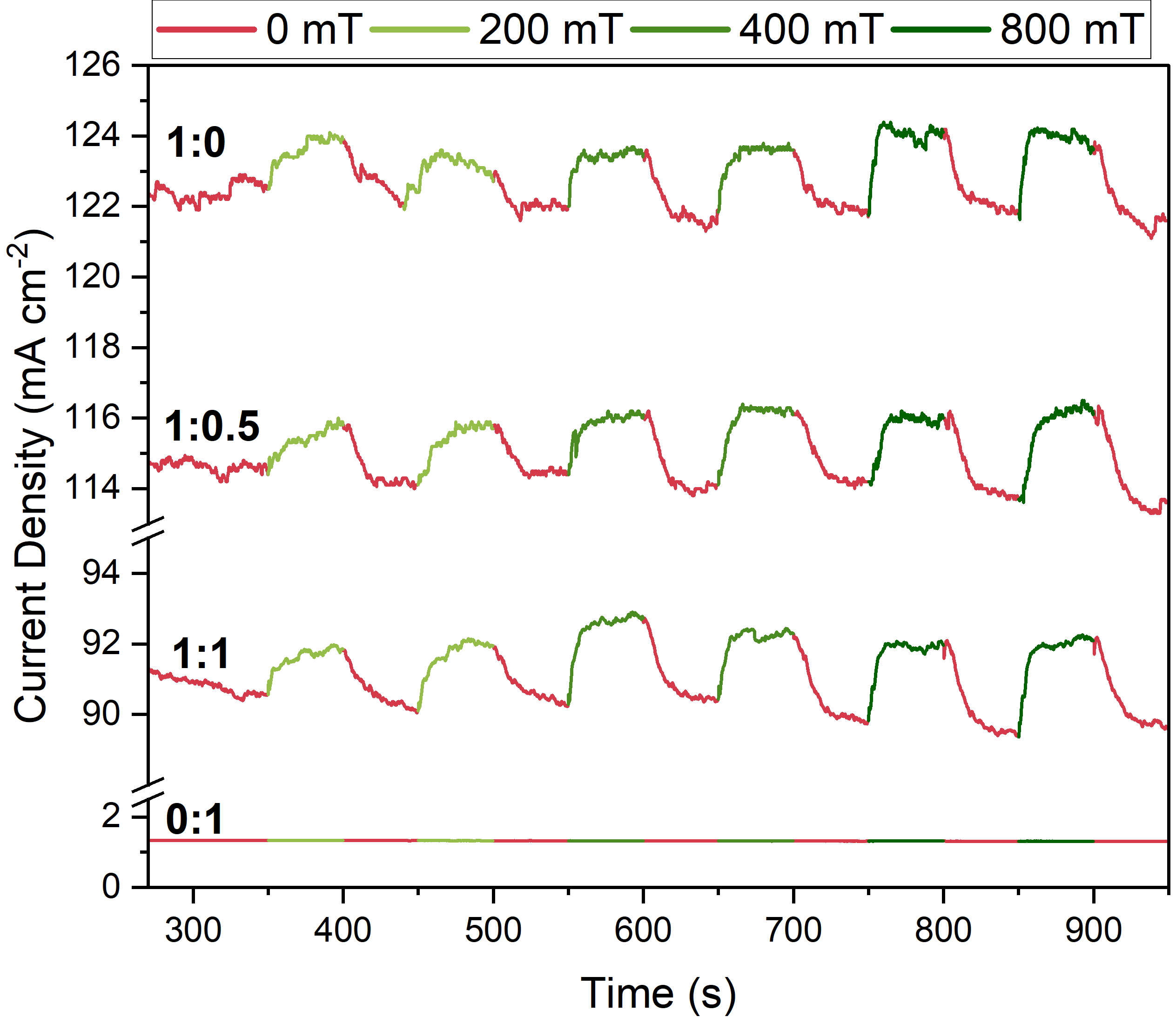


Figure S24. PMCA plots (2 V, 0^o^ orientation) of Co_3_O_4_, CoBa_0.5_, CoBa_1.0_, and BaFe_12_O_19_.


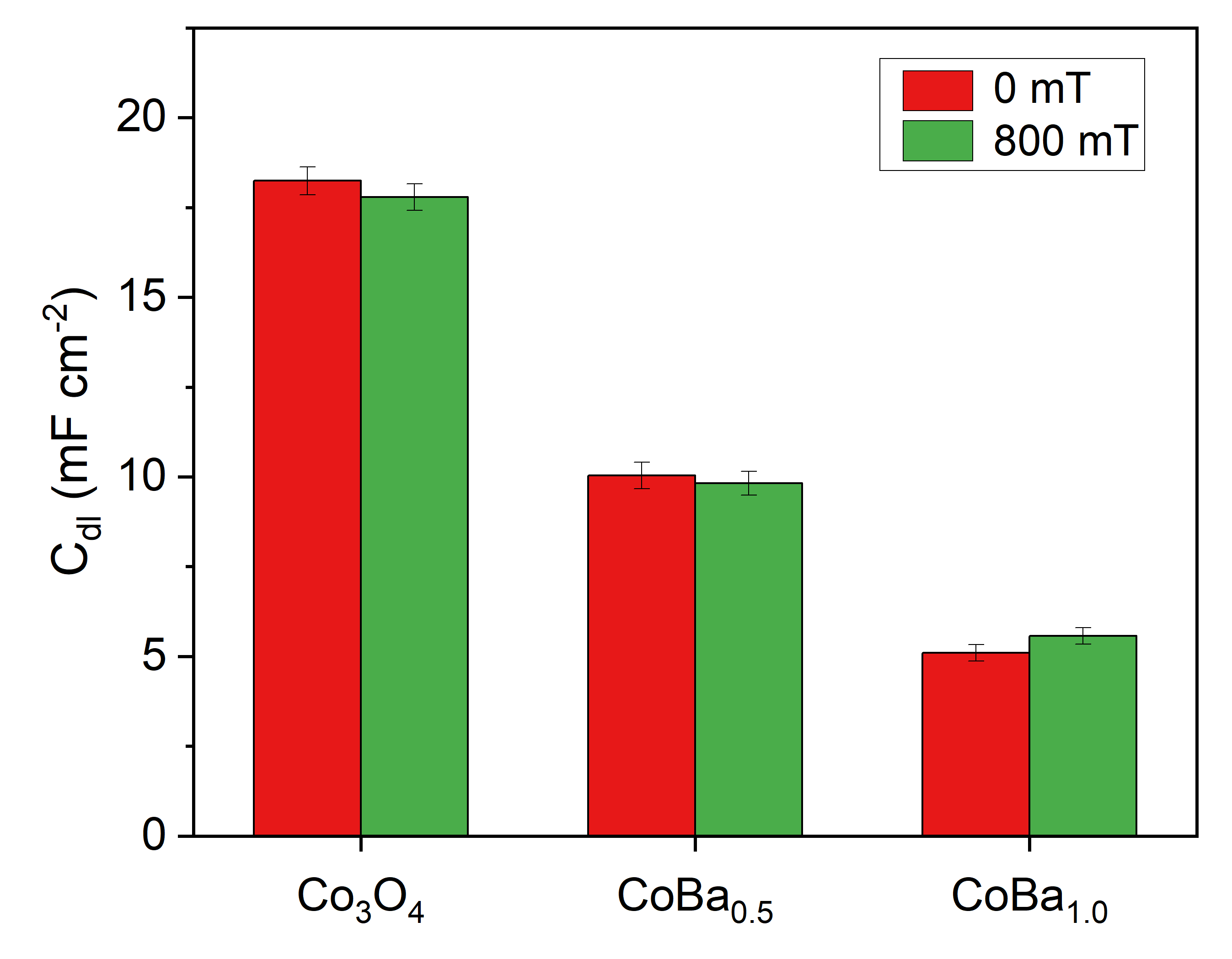
**Figure S25.** Double layer capacitance (directly proportional to ECSA) for Co_3_O_4_, CoBa_0.5_, and CoBa_1.0_ under and 800 mT.


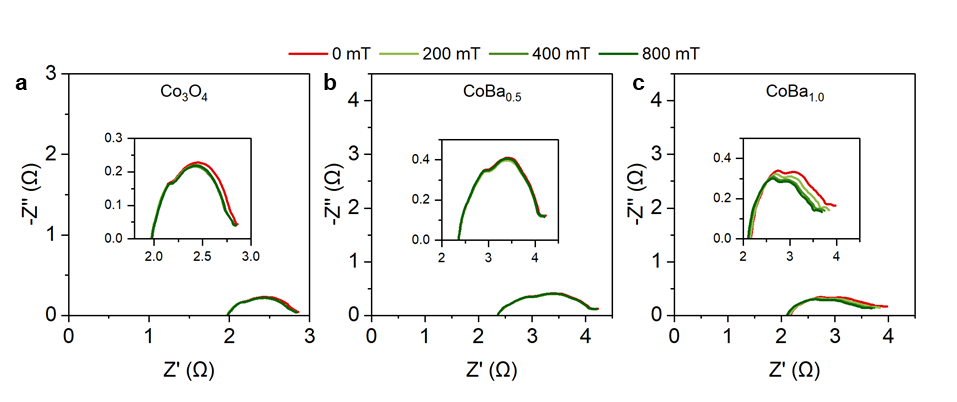


Figure S26. EIS plots taken at 1.8 V vs RHE for *a*) Co_3_O_4_, *b*) CoBa_0.5_, and *c*) CoBa_1.0_ under 0, 200, 400, and 800 mT fields.


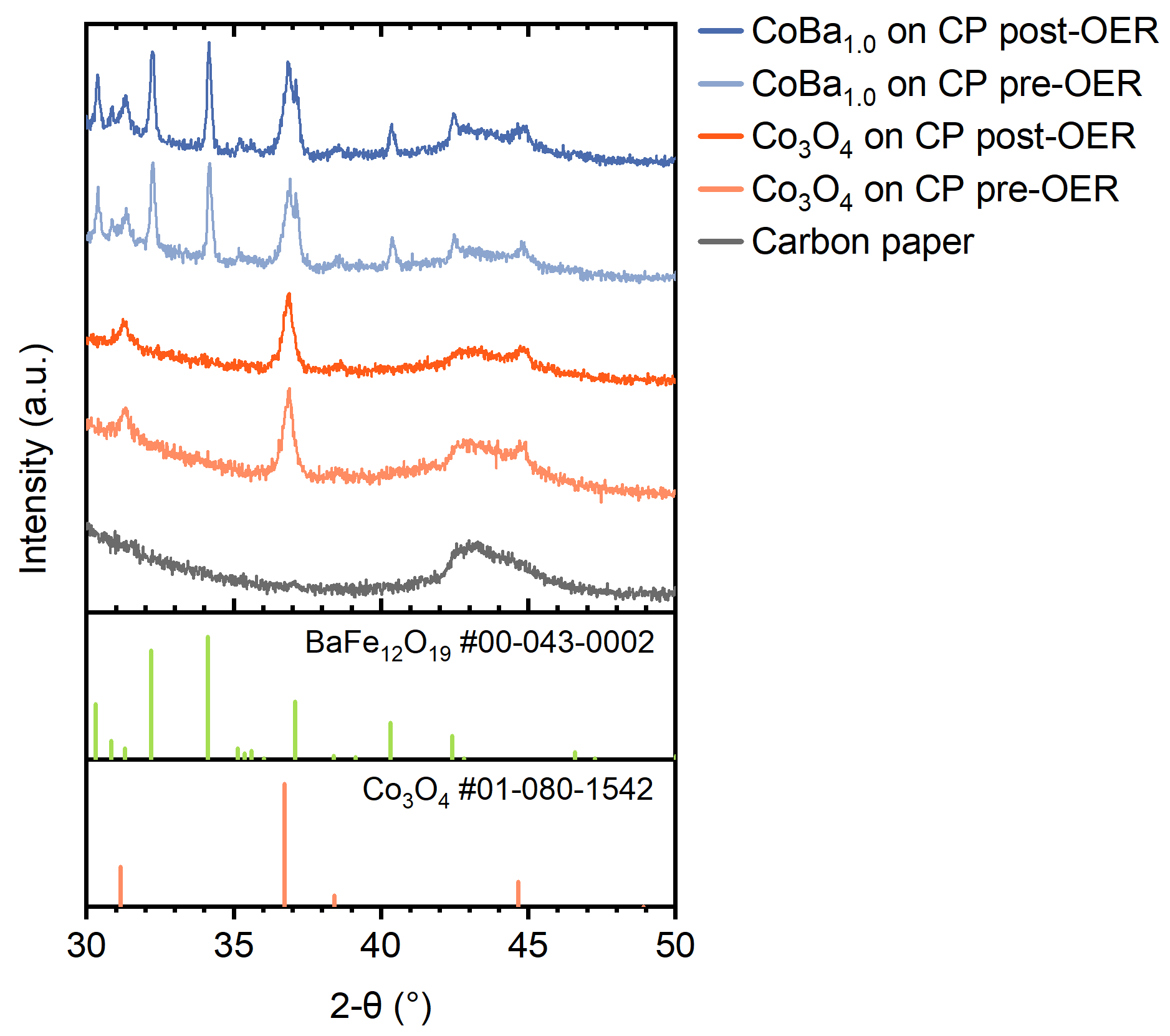


Figure S27. XRD patterns obtained for carbon paper (CP) substrate, and Co_3_O_4_ and CoBa_1.0_ catalysts before and after reaction, showing no change in XRD patterns. X-scale was adjusted to focus on the 30-50 2-θ region as includes the main peaks for both powders.


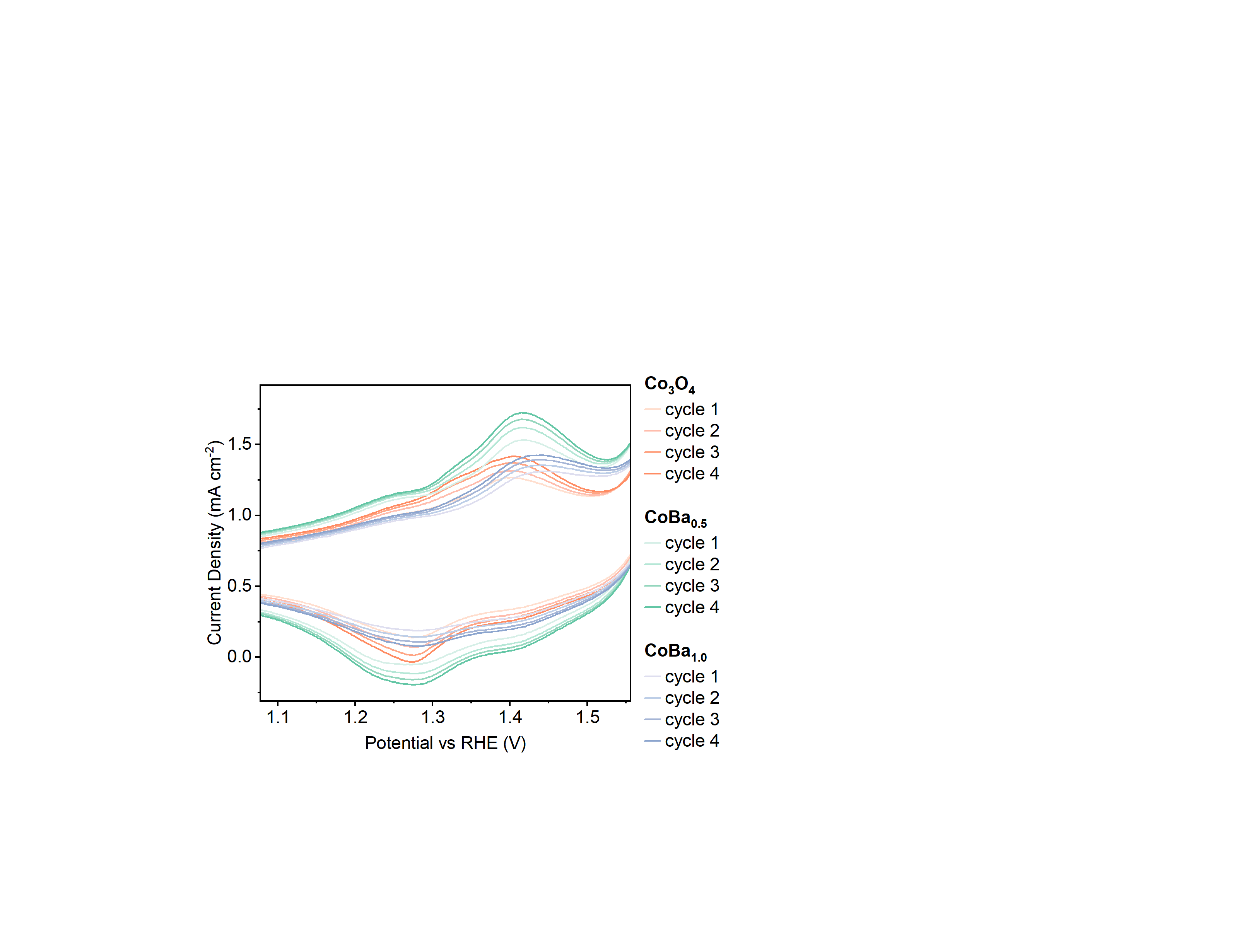


Figure S28. CV plots conditioning, cycles 1 to 4, for Co_3_O_4_, CoBa_0.5_, and CoBa_1.0_. Focussed on the redox region.


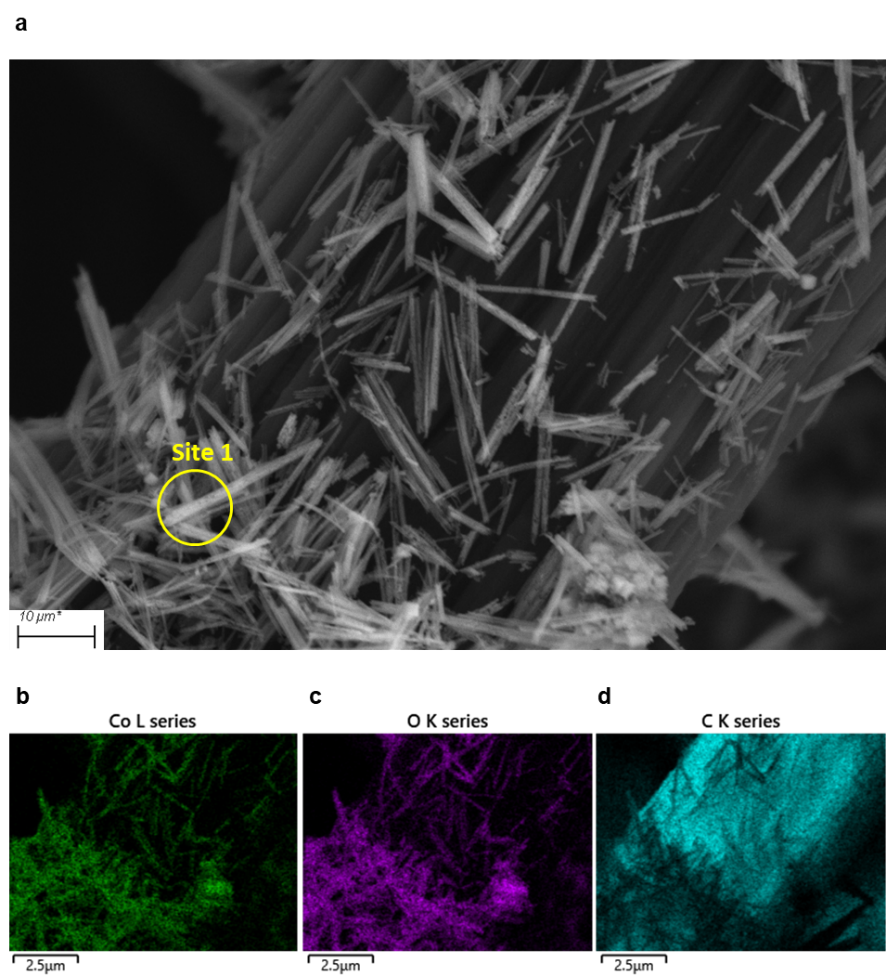


Figure S29. *a)* SEM image of Co_3_O_4_ deposited on carbon paper pre-OER. EDS mapping of *b)* Co, *c)* O and *c)* C content.


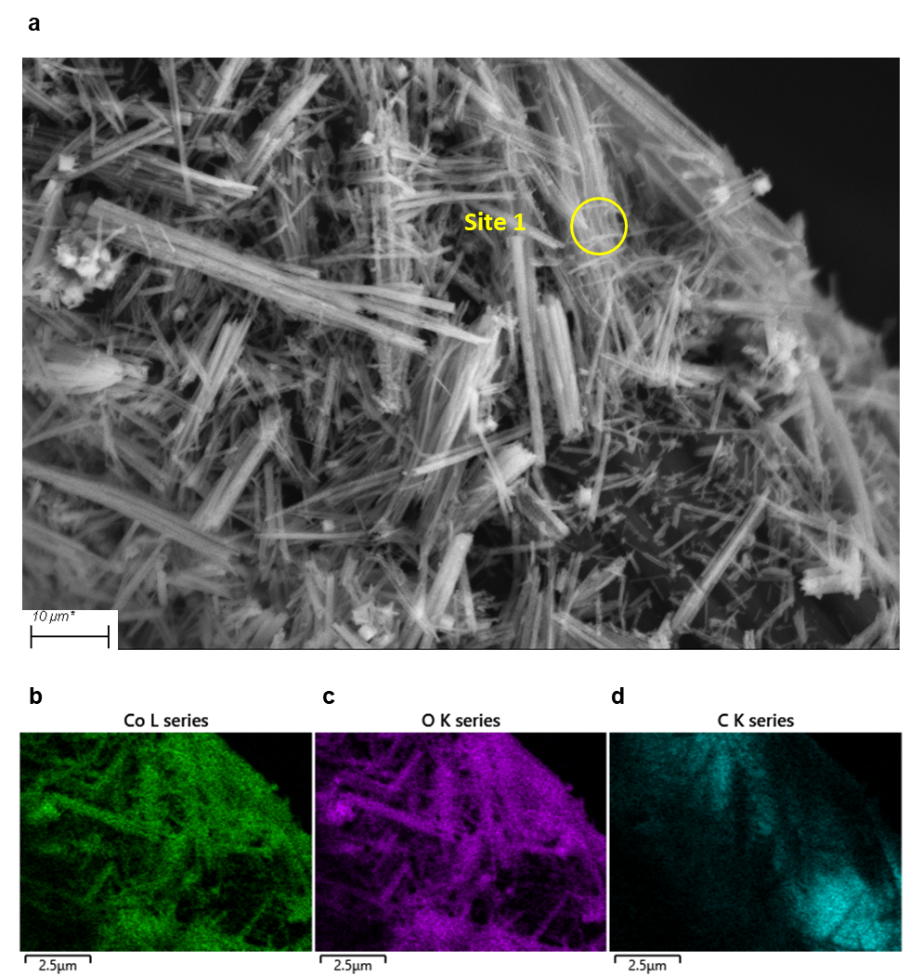


Figure S30 *a)* SEM image of Co_3_O_4_ deposited on carbon paper post-OER. EDS mapping of *b)* Co, *c)* O and *d)* C content.


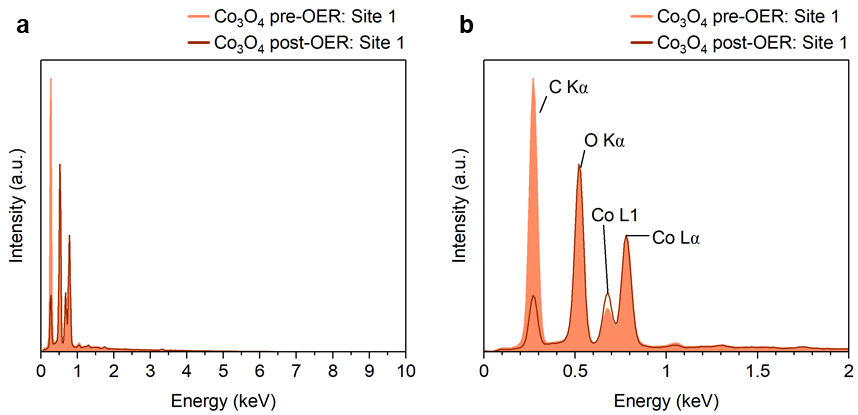


Figure S31 Figure SX. EDS plots of Co_3_O_4_ deposited on carbon paper pre- and post-OER: *a)* full scan and *b)* zoomed in on region 0-2 keV.


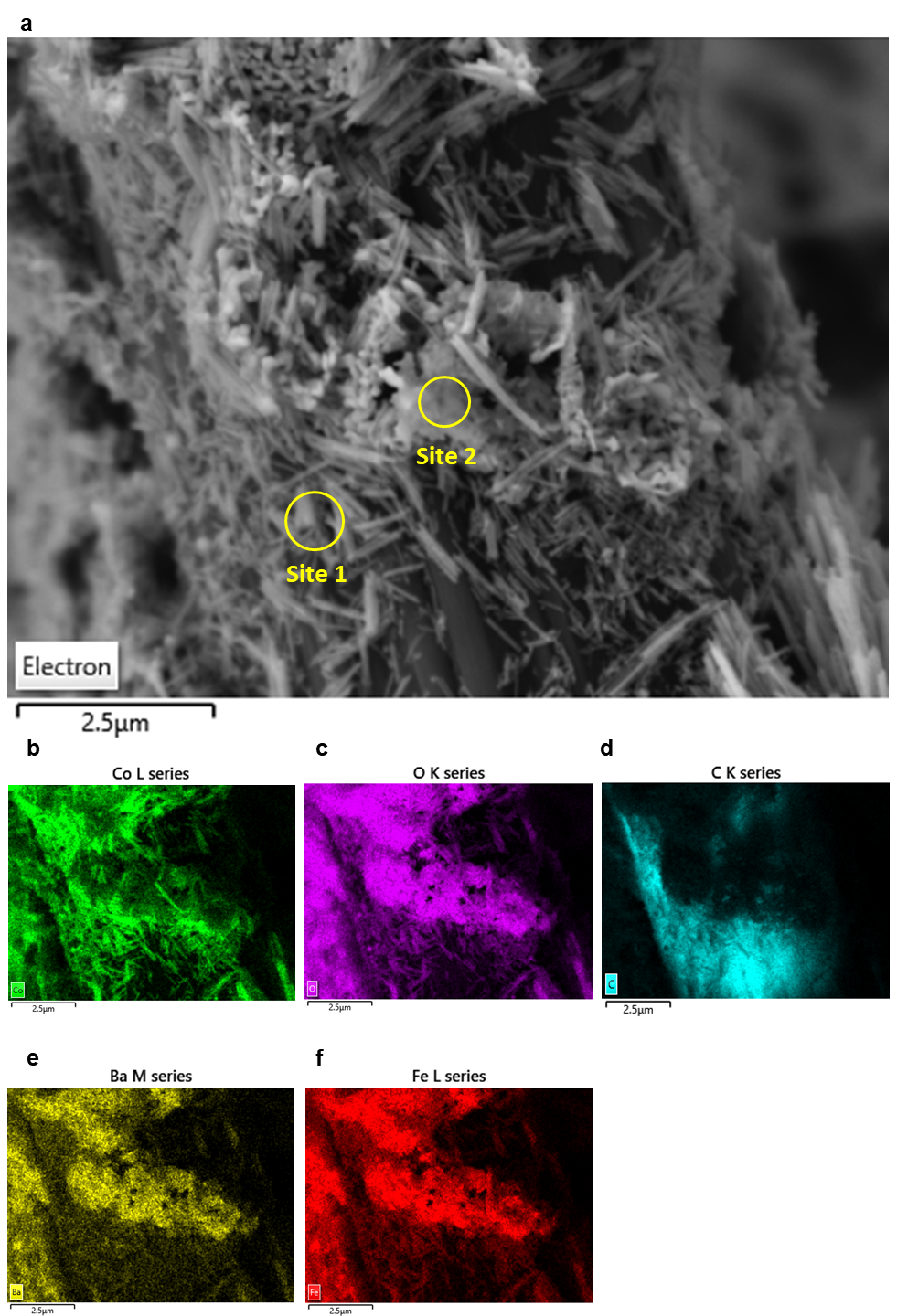


Figure S32. a) SEM image of CoBa_1.0_ deposited on carbon paper pre-OER. EDS mapping of *b)* Co, *c)* O, *d)* C, *e)* Ba and *f)* Fe content.
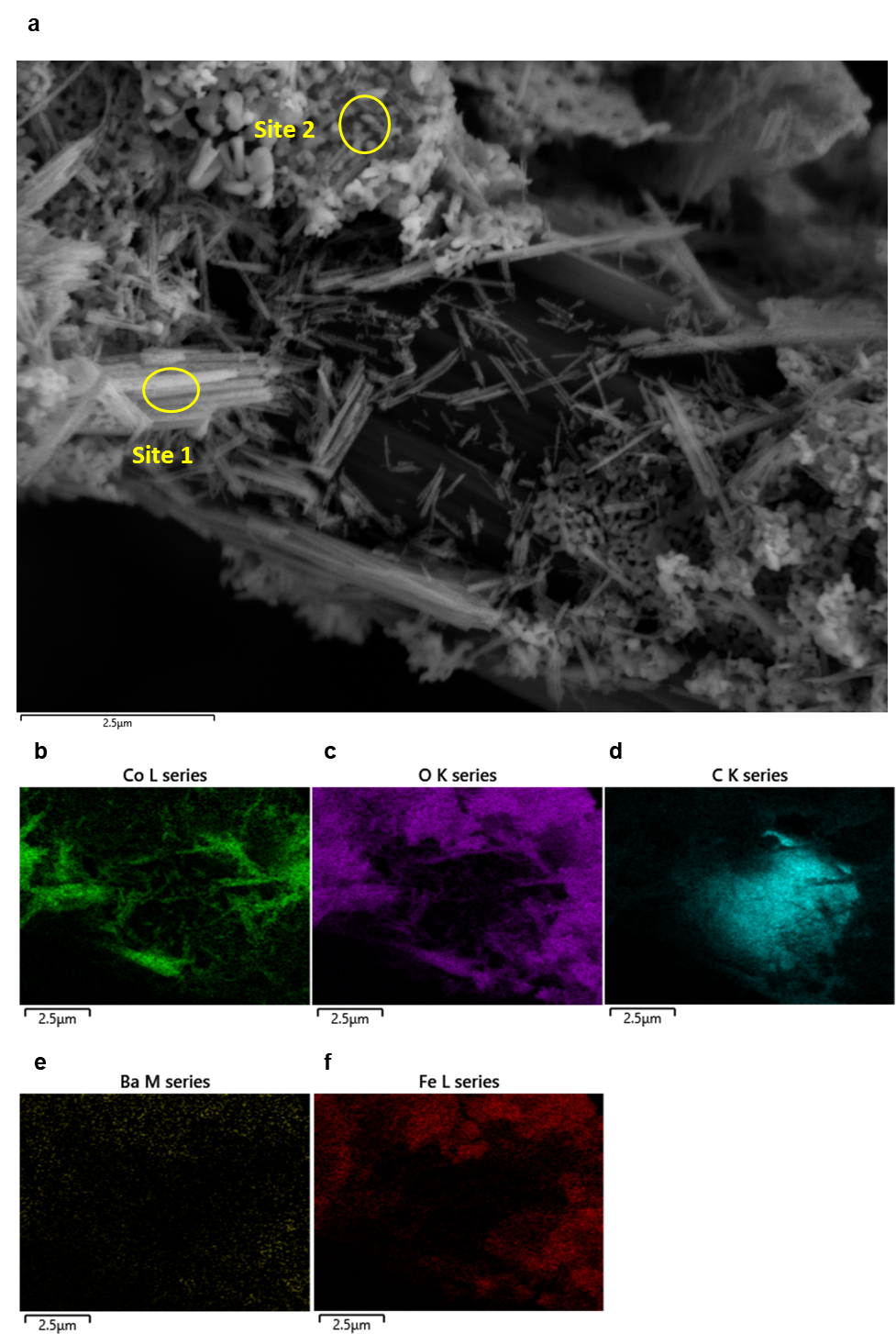


Figure S33. *a)* SEM image of CoBa_1.0_ deposited on carbon paper post-OER. EDS mapping of *b)* Co, *c)* O, *d)* C, *e)* Ba and *f)* Fe content.


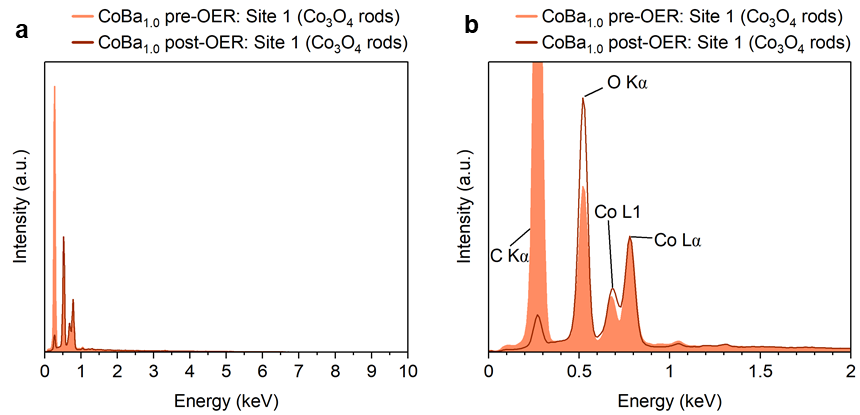


Figure S34. EDS plots of CoBa_1.0_ deposited on carbon paper pre- and post-OER. Sites with Co_3_O_4_ rods are imaged: *a)* full scan and *b)* zoomed in on region 0-2 keV.


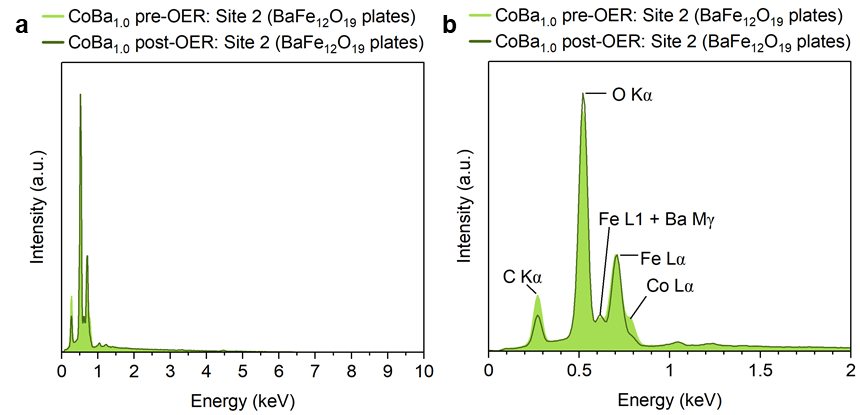


Figure S35. EDS plots of CoBa_1.0_ deposited on carbon paper pre- and post-OER. Sites with BaFe_12_O_19_ plates are imaged: *a)* full scan and *b)* zoomed in on region 0-2 keV.


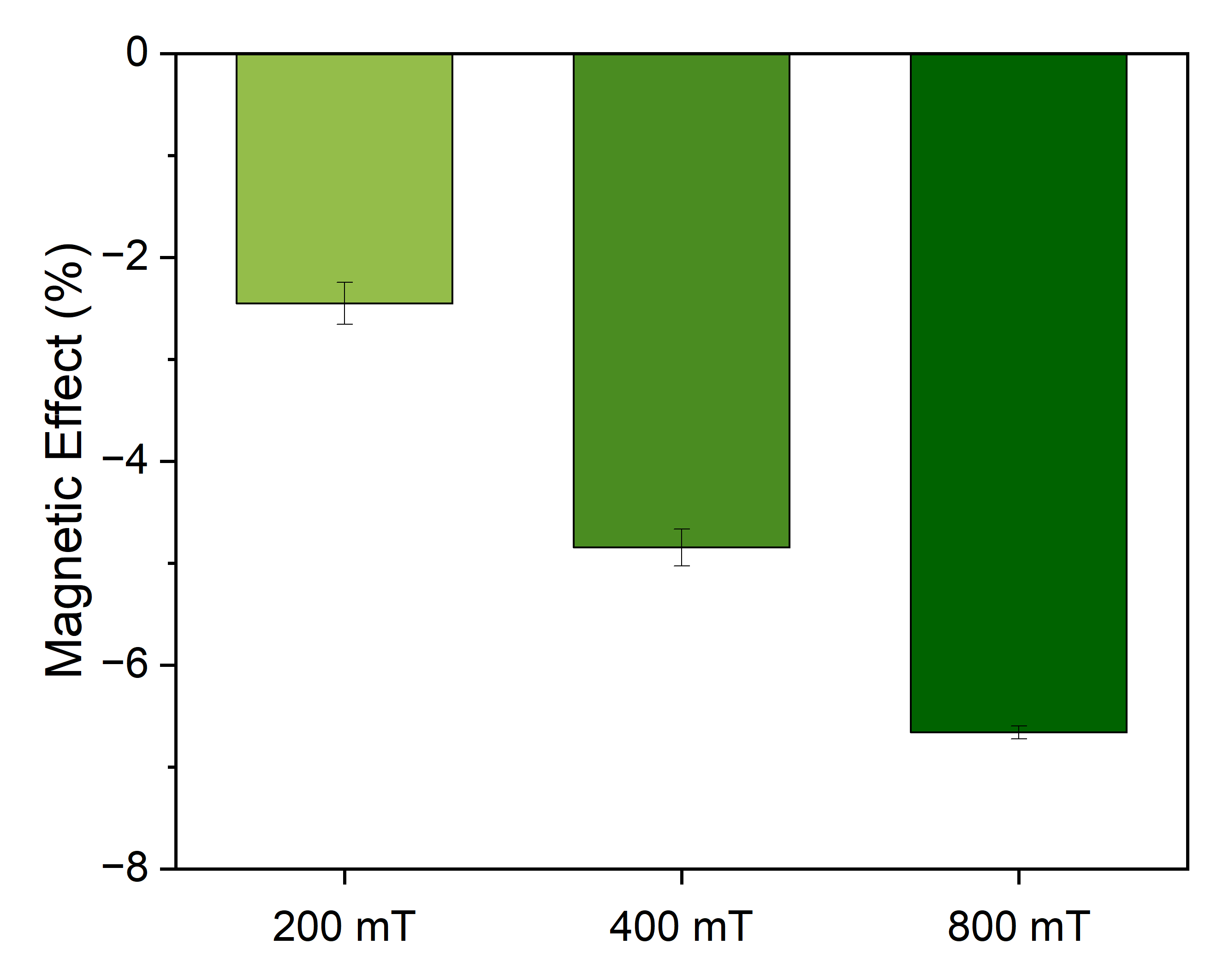


Figure S36. Percentage magnetic effect of carbon paper calculated from PMCA measurements at 0^o^ orientation.


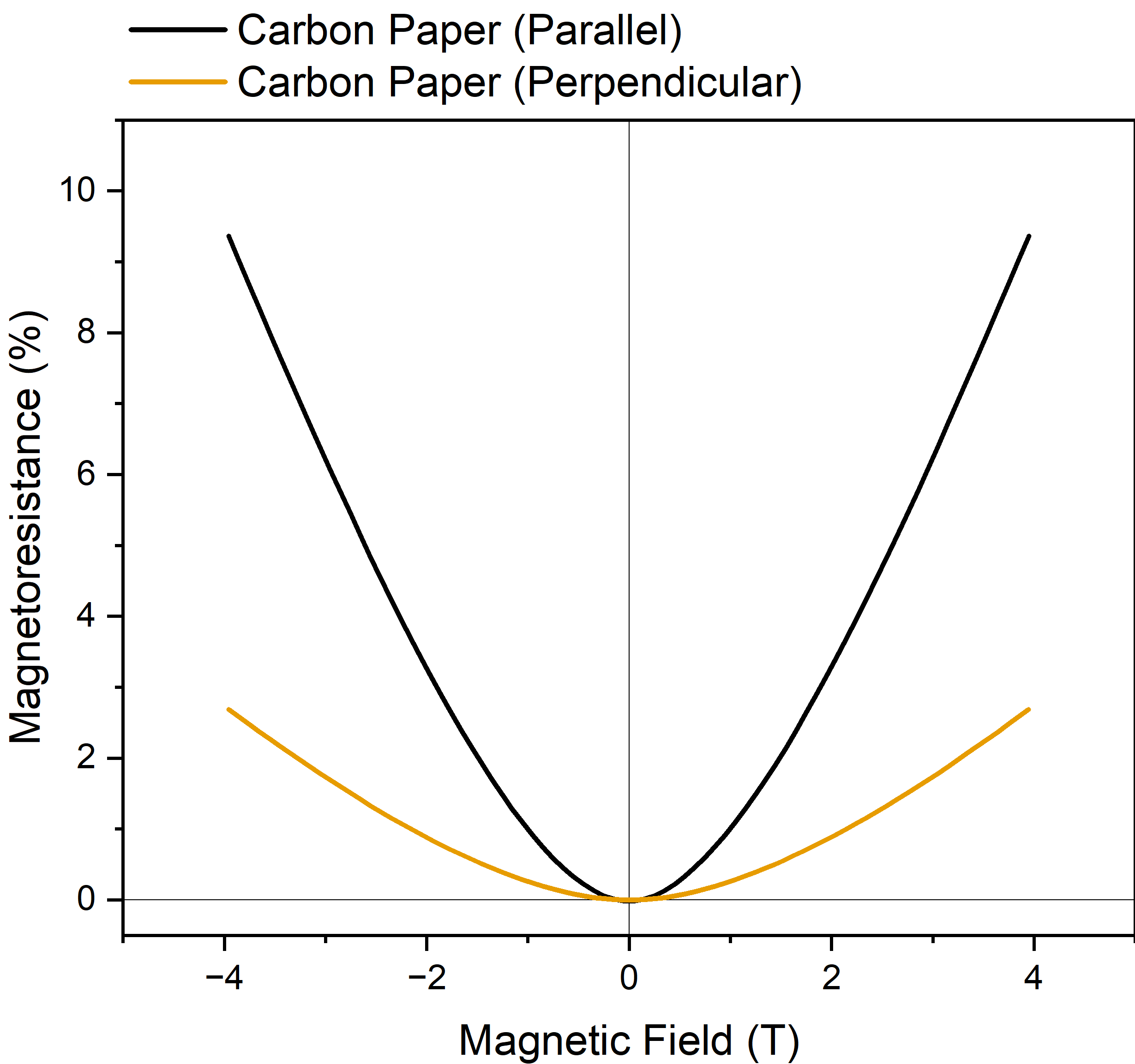


Figure S37. Magnetoresistance plots of commercial carbon paper. Where the magnetic field is perpendicular to the direction of current flow (5 mA) and parallel orientation refers to out-of-plane alignment to the electrode surface and perpendicular orientation refers to in-plane alignment. The data was symmetrized to remove Hall contributions.


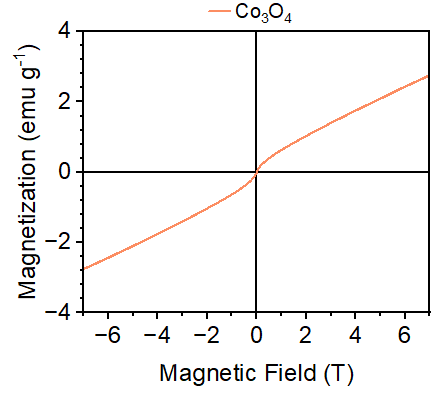


Figure S38. M v H curve of Co_3_O_4_.


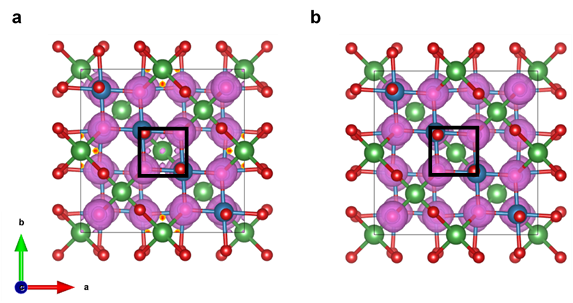


Figure S39. Computed electron localization function for *a)* AFM and *b)* FM for Co₃O₄ with isovalue = 0.6. (Oxygen: red, Co^2+^: green, Co^3+^: blue)
